# Supramolecular macrocycles reversibly assembled by Te^…^O chalcogen bonding

**DOI:** 10.1038/ncomms11299

**Published:** 2016-04-19

**Authors:** Peter C. Ho, Patrick Szydlowski, Jocelyn Sinclair, Philip J. W. Elder, Joachim Kübel, Chris Gendy, Lucia Myongwon Lee, Hilary Jenkins, James F. Britten, Derek R. Morim, Ignacio Vargas-Baca

**Affiliations:** 1 Department of Chemistry and Chemical Biology, McMaster University, 1280 Main Street West, Hamilton, Ontario, Canada L8S 4M1

## Abstract

Organic molecules with heavy main-group elements frequently form supramolecular links to electron-rich centres. One particular case of such interactions is halogen bonding. Most studies of this phenomenon have been concerned with either dimers or infinitely extended structures (polymers and lattices) but well-defined cyclic structures remain elusive. Here we present oligomeric aggregates of heterocycles that are linked by chalcogen-centered interactions and behave as genuine macrocyclic species. The molecules of 3-methyl-5-phenyl-1,2-tellurazole 2-oxide assemble a variety of supramolecular aggregates that includes cyclic tetramers and hexamers, as well as a helical polymer. In all these aggregates, the building blocks are connected by Te^…^O–N bridges. Nuclear magnetic resonance spectroscopic experiments demonstrate that the two types of annular aggregates are persistent in solution. These self-assembled structures form coordination complexes with transition-metal ions, act as fullerene receptors and host small molecules in a crystal.

## Abstract (web summary)

Similarly to halogen bonding, the heavier chalcogens are capable of forming supramolecular links with electron rich sites. Here, the authors show that these forces can allow the formation of well-defined cyclic structures that are stable in solution and are capable of forming host-guest complexes.

## Graphical abstract

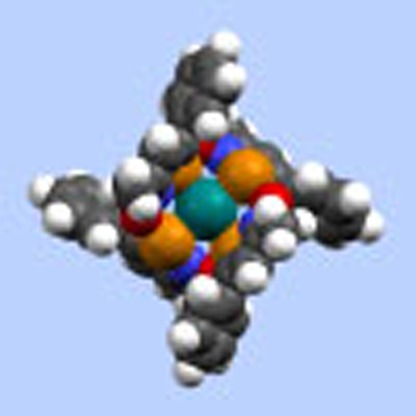

One of the most remarkable developments in supramolecular chemistry in the last two decades is the evolution of halogen bonding^1^,^2^ from being an intriguing structural feature to becoming a powerful tool in crystal engineering^3^,^4^,^5^,^6^, which is also applicable to systems and processes as diverse as luminescent^7^ and non-linear optical^8^ materials, photo-patterning of surfaces^9^, the assembly of fractal patterns from molecular building blocks^10^, supramolecular gelation^11^, organocatalysis^12^, macromolecular alignment at macroscopic scale^13^, anion recognition^14^, transmembrane anion transport^15^,^16^ and mimicking the activity of the deiodinase enzyme^17^.

Our current understanding attributes such interactions to the depletion of electron density in a region of space surrounding the nucleus of a halogen atom in a molecule (termed a sigma hole)^11^, which consequently is attracted to lone-pairs and *π* clouds of electrons. This electrostatic factor is supplemented by polarization of the electron density—which in a strong case would be modelled by the mixing of electron donor and acceptor orbitals—and electron correlation manifested in the London dispersion force. Those stabilizing contributions are countered by the Pauli repulsion and the balance results in interaction energies of about 5–70 kJ mol^−1^ and preference for a linear *R*-*X*^**…**^:*B* geometry (*X* is a halogen atom and *B* a Lewis base)^18^,^19^,^20^. The conditions that give rise to each of those stabilizing factors are common to molecules that contain heavy elements from other p-block families^18^,^21^,^22^,^23^,^24^,^25^. While it is long-recognized^26^,^27^,^28^ that intra- and intermolecular short interatomic contacts are pervasive in structural main-group chemistry, terms such chalcogen and pnictogen bonding have been recently suggested by analogy to the halogen case. There are indeed common traits to all these interactions; for instance, the trends in ratio of interatomic distance to sum of van der Waals radii denote a correlation of interaction strength with the mass of the p-block element and enhancement by electronegative substituents^29^,^30^,^31^. However, one important difference is that atoms of group-16 (refs 32, 33) and 15 (refs 34, 35) elements can engage in up to two and three concurrent supramolecular interactions, respectively^36^,^37^.

The potential of chalcogen-centered interactions in supramolecular chemistry is illustrated by two well-studied molecular families: the dichalcogena alkynes, which consistently crystalize in tubular structures assembled by chalcogen–chalcogen interactions^38^,^39^,^40^, and the 1,2,5-chalcogenadiazoles, in which two pairs of antiparallel chalcogen–nitrogen interactions per molecule tend to build infinite ribbons. The latter structures are of interest because of their charge transport properties^41^ and, through moderate steric repulsion, can be distorted to induce non-linear optical properties or chromotropism^42^,^43^,^44^. The auto-association of chalcogenadiazoles is amenable to combination with metal-ion coordination and hydrogen bonding^45^,^46^. In spite of their charge, *N*-alkylated chalcogenadiazolium cations do associate in the solid state and, according to electrochemical data, the tellurium derivatives may also be associated in solution^47^.

Large assemblies (gels, supramolecular polymers and crystals) provide tangible demonstration of the power of these supramolecular interactions but comparatively less has been investigated at the other end of the size spectrum: small, discrete aggregates of a few molecules held together by supramolecular bonds. In the halogen-bonding case, I^**…**^N interactions have been employed in the construction of molecular capsules^48^ from a pair of complementary molecules; the assembly of such structures in solution was recently demonstrated^49^,^50^.

In the chalcogen case, the crystal of 3-methyl-5-(1,1-dimethylethyl)-1,2-tellurazole 2-oxide (**1a)**^51^ features a tetramer spontaneously assembled by short Te^**…**^O interactions, its arrangement is specially intriguing because it evokes a macrocycle. However, until now it has been unclear whether such an aggregate would be stable enough to function in the same way as do molecules from the vast category of supramolecular building blocks that encompasses crown ethers, polyazacycloalkanes, tetrapyrroles, phthalocyanines, calixarenes, cyclodextrins, cucurbiturils and cyclophanes. If so, building macrocycles by the spontaneous addition of molecular building blocks would be a uniquely convenient approach because, in general, the synthesis of macrocycles is laborious and low-yielding—even when template methods are used—with notable exceptions such as the recently synthesized macrocyclic cyanostar^52^. We have investigated in detail the stability of the cyclic aggregates of iso-tellurazole *N*-oxides in solution and probed their chemical behaviour. Here we report that indeed these assemblies are persistent in solution and display properties of actual macrocyclic molecules in their ability to coordinate transition-metal ions, form adducts with fullerenes and host small species in the solid state.

## Results

### Preparation and structural studies

**Figure 1:**
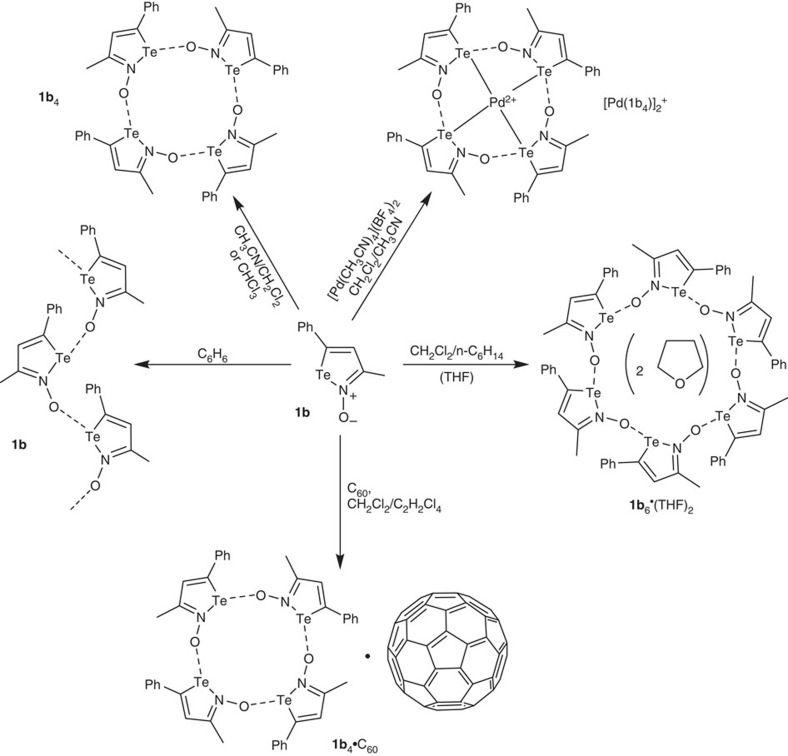
Summary of supramolecular species derived from 1b. Supramolecular species formed by auto-association of **1b**, alone or in combination with a Pd(II) salt or C_60_.

**Figure 2:**
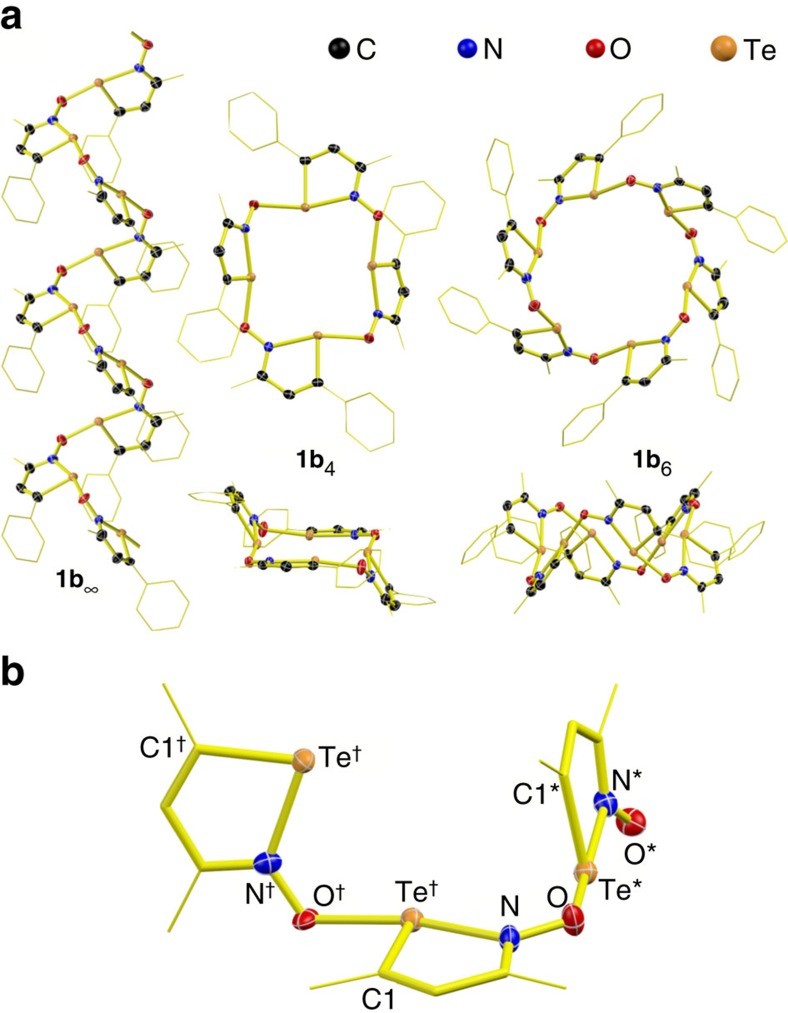
Crystallographically characterized aggregates of 1b. (**a**) ORTEPs of the aggregates observed in the crystal structures of 3-methyl-5-phenyl-isotellurazole-*N*-oxide, **1b**. While **1b**_∞_ is contained in 3(**1b**)·(C_6_H_6_), the macrocyclic tetramer crystallizes in a solvent-free polymorph and the hexamer forms the co-crystal 12(**1b**)·(CH_2_Cl_2_). (**b**) Detail of the structure of **1b**_∞_ displaying the relative orientations of the iso-tellurazole planes and the labelling sequence used in the discussion. Displacement ellipsoids are plotted at 75% probability in all cases. For clarity, hydrogen atoms are omitted, the phenyl and methyl groups are portrayed using a wireframe representation and are partially hidden in **b**.

Improvements from literature procedures^51^,^53^ afford the iso-tellurazole *N*-oxides **1a** and **1b** in good yields; these products are remarkably stable in air and tolerant of moisture. Depending on the solvent used for crystallization, 3-methyl-5-phenyl-iso-tellurazole *N*-oxide (**1b**) crystallizes in a remarkable variety of polymorphs, all the structures in Fig. 1 were identified by single-crystal X-ray diffraction. In each case, the morphology of the whole sample indicates that only one phase is reproducibly obtained. The unit cells of several polymorphs include solvent molecules and, while the solvent likely influences packing efficiency, the molecules of **1b** are all associated to each other only. The aggregates observed in the crystals (Fig. 2a) include an infinite spiral chain (**1b**_∞_) and cyclic tetra- (**1b**_4_) and hexamers (**1b**_6_). In every case, consistently with the σ-hole/donor–acceptor model, the oxygen atom of one molecule is bound to the tellurium atom of another, always trans to the nitrogen atom and to distances that span 2.171(3) to 2.242(1) Å. These are comparable to the 2.299(2) Å measured in **1a**_4_ (ref. 51) and are slightly longer than the 2.122(1) Å of the axial bonds in β-TeO_2_ (ref. 54). The Te–N distances (2.197(2) to 2.258(2) Å) are slightly longer than those measured for other single bonds between these elements. In each crystal, the geometry around the chalcogen approximates a T-shape, with N–Te–O^†^, N–Te–C and O–N–Te average angles of 165.3°, 76.0° and 126.9°, respectively. Individual iso-tellurazole heterocyles are planar, with bond distances consistent with localized single and double bonds. Adjacent iso-tellurazole rings tend to lay perpendicular to each other (Fig. 2b) with inter-planar angles ranging from 60° to 83°.

**Figure 3:**
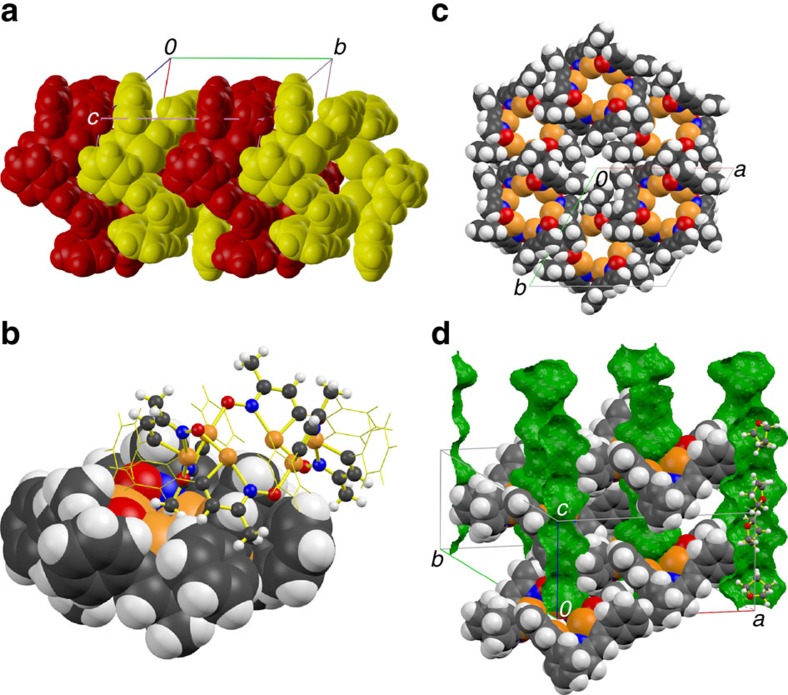
Detail of the crystalline structures that feature the hexamer 1b_6_. From the crystal of composition 12(**1b**)·(CH_2_Cl_2_): (**a**) stacking of the macrocycles, (**b**) interaction of the methyl group with the cavity of a neighbouring ring. From the crystal of composition 3(**1b**)·(C_4_H_8_O): (**c**) packing of a layer in the (0,0,1) plane, (**d**) detail of the crystal structure, highlighting the channels, as well as the location and three orientations of the THF molecules. Panels **c** and **d** are simplified for clarity; after ruling out twinning, larger unit cells and less symmetric space groups, the best approximate model features three distinct orientations for the phenyl groups but only one orientation for the C_3_NTe ring. The THF molecules were treated as rigid groups.

Crystallization from benzene yields a phase of composition 3(**1b**)·(C_6_H_6_), which is built by infinite spiral chains (**1b**_∞_) coiling in alternating directions along *b* with a periodicity of 3. However, the P2_1_2_1_2_1_ space group contains no ternary screw axis, thus the unit cell contains three crystallographically distinct molecules. The macrocyclic tetramer **1b**_4_ is formed in non-solvated crystals obtained from CHCl_3_ or by layering acetonitrile over a CH_2_Cl_2_ solution. Its structure resembles that of **1a**_4_ but there are important differences: the structure of the phenyl derivative approaches a chair conformation, has *C*_*i*_ symmetry and is built by two crystallographically independent molecules while the geometry of the *t*-Bu aggregate corresponds to a boat conformer, belongs to the *S*_*4*_ point group and the four constituting **1a** molecules are all related by symmetry^51^. There are two distinct trans-annular Te–Te distances in **1b**_4_, 5.5895(2) and 5.3043(2) Å. Crystallization from THF and a hexanes/CH_2_Cl_2_ mixture produces crystals of compositions 3(**1b**)·(C_4_H_8_O) and 12(**1b**)·(CH_2_Cl_2_), respectively. Both phases contain macrocyclic hexamers. In the latter case, the crystallization solvent occupies voids external to two crystallographically distinct macrocycles, each built from three molecular units that are unique by symmetry. Packing distorts the macrocyle, thus there are three different trans-annular Te–Te distances for each ring: 7.117(1), 7.300(1) and 7.544(1) Å in one case and 7.151(1), 7.227(1) and 7.692(1) Å in the other. As the macrocycles in this crystal stack along *b* (Fig. 3a) a methyl group of one hexamer extends towards the cavity of the neighbouring macrocycle (Fig. 3b). In contrast, the crystal that contains THF packs in a hexagonal lattice (Fig. 3c); the macrocyclic aggregate is built by six equivalent molecular units of **1b** and the trans-annular Te–Te distances are all 7.638(2) Å. The macrocycles pack in a layer and a second cavity is flanked by the phenyl groups. Vertical stacking of the layers in an ABA sequence alternates the two types of cavity-forming tubular channels (Fig. 3d). THF molecules disordered in three different orientations sit in the macrocycle cavities, above and below the plane defined by the chalcogens. Although these channels are small, the crystals slowly loose solvent and become opaque.

### Evidence of persistent auto-association in solution

**Figure 4:**
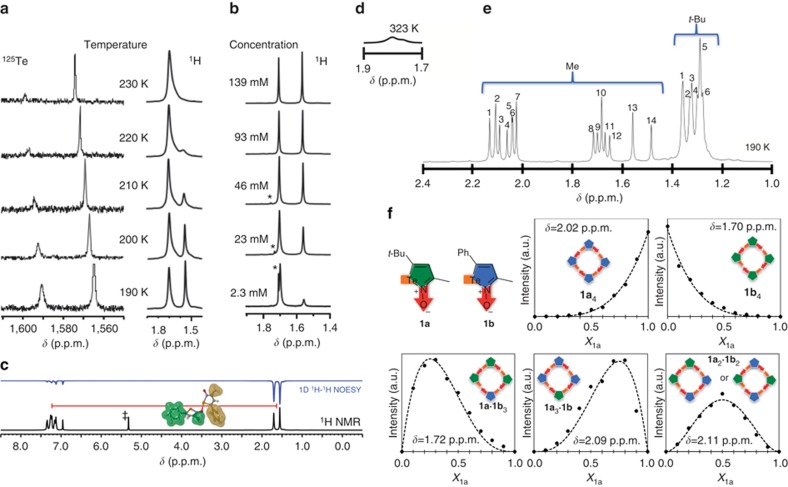
NMR Investigations of the auto-association of 1 in solution. NMR (500 MHz) spectra of **1b** solutions in CDCl_3_: (**a**) ^125^Te and ^1^H at 74 mmol l^−1^ and variable temperature; (**b**) ^1^H at 190 K as a function of concentration; *denotes the resonance of a trace amount of H_2_O. (**c**) ^1^H-NMR and 1D-NOESY spectra of **1b** in CD_2_Cl_2_ at 190 K; the over-imposed structure portrays the van der Waals surfaces of the methyl and phenyl groups of each molecule in a pair modelled with coordinates extracted from the crystal structures of **1b**_4_; ^‡^denotes the resonance of residual CHDCl_2_. ^1^H-NMR (600MHz) from the equimolar mixture of **1a** and **1b**: (**d**) methyl resonances above room temperature, (**e**) methyl and *t*-butyl resonances at low temperature. (**f**) evolution of selected methyl ^1^H-NMR lines as a function of composition (molar fraction of **1a**) of the mixtures, dashed lines correspond to the calculated abundance of each type of aggregate.

In spite of the large molecular mass, the electrospray mass spectrum (Supplementary Fig. 1) displays isotopic patterns characteristic of aggregates [**1b**_n_-H]^+^ (*n*=1–7). Given the apparent strength of the Te^**…**^O supramolecular interactions, it became of interest to probe the aggregation of iso-tellurazole oxides in solution with nuclear magnetic resonance (NMR) spectroscopy. At room temperature, the ^125^Te NMR spectrum of **1b**, measured in CH_2_Cl_2_, displays only a single line. However, a second line appears on cooling and grows in intensity at the expense of the first (Fig. 4a). These changes are fully reverted when room temperature is restored and are paralleled by those in the ^1^H-NMR spectrum, albeit with some differences. For example, the lines of the methyl resonances coalesce at 230 K in the 500 MHz spectrum. Furthermore, the relative intensities of the methyl lines are dependent on concentration (Fig. 4b). At low temperature, negative nuclear Overhauser effect (NOE) is observed between the ^1^H nuclei of the methyl and phenyl or *t*-butyl groups of **1b** (Fig. 4c) and **1a** (Supplementary Fig. 2), respectively. The separation between the ^1^H nuclei of these pendant groups in the individual molecules of **1** is too large for NOE (>5 Å). However, the crystal structures show that the Te^**…**^O interactions bring the substituents of neighbouring molecules to shorter distances (<3 Å).

### NMR scrambling experiments

Mixtures of **1a** and **1b** were investigated by ^1^H-NMR to further probe the structure of their aggregates in solution. At room temperature, the spectrum of the 1:1 mixture displays a very broad band (1.9–1.6 p.p.m.) for the methyl resonances, this feature is more clearly discernible at 323 K (Fig. 4d), these observations are indicative of intermolecular association and exchange. The spectrum with the best resolution of resonances from this mixture was acquired at 190 K using a 600 MHz instrument; in these conditions 14 lines are observed for the methyl and 6 for the *t*-butyl resonances (Fig. 4e). The methyl lines in the ^1^H-NMR spectra of mixtures of variable composition but constant total concentration were classified by their behaviour as a function of molar fraction (Fig. 4f). It was possible in this way to identify the patterns characteristic of cyclic tetramers: resonances that continuously increase or decrease in intensity following a fourth-degree polynomial trajectory for the homogeneous aggregates; lines with one maximum each at molar fractions 0.25 or 0.75 that correspond to the mixed tetramers of 1:3 and 3:1 composition; as well as lines with a maximum at 0.5 that correspond to the 2:2 stoichiometry. Lines with a more complex behaviour would result from the superposition of more than one resonance.

As this interpretation attributes the resonance at 1.70 p.p.m. to the methyl protons of the tetramer **1b**_4_, it became possible to explain the effect of concentration on the ^1^H-NMR spectrum of **1b** (Fig. 4b) as the result of further aggregation (equation (1)). The stoichiometric coefficient (*n*) and equilibrium constant (*K*, equation (2)) of the process were determined from the intensities (*I*) of the lines (relative to the resonance of the residual proton on the solvent) as a function of concentration (equation (3), Supplementary Fig. 3a). The fitted stoichiometric coefficient *n*=1.53±0.02 implies that the resonance at 1.56 p.p.m. belongs to the methyl protons of the hexamer. At 190 K, the equilibrium constant is therefore *K*=0.28±0.01 dm^1.5^ mol^−0.5^; the corresponding van't Hoff analysis (Supplementary Fig. 3b) yielded Δ*H*=−16±1 kJ mol^−1^ and Δ*S*=−88±6 J mol^−1^K^−1^. However, diffusion-ordered spectroscopy (DOSY) experiments were unable to provide the hydrodynamic radii and mass of the aggregates because the magnetization decay during diffusion did not follow simple Gaussian profiles, which likely is a consequence of the equilibrium between tetramer and hexamer.


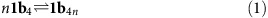



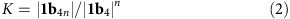



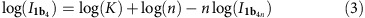


### Computational modelling

**Figure 5:**
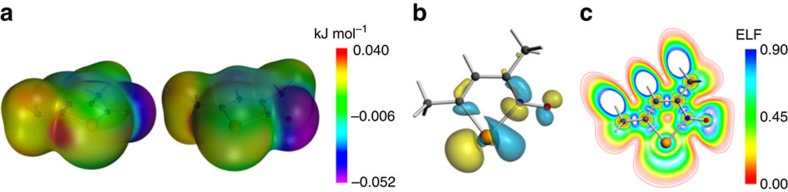
The aggregation of 1 originates in its electronic properties. (**a**) Two views of the map of electrostatic potential for a molecule of **1c**; (**b**) LUMO of **1c**; (**c**) contour plot of the electron localization function in the iso-tellurazole plane.

The relative stabilities of the oligomeric structures formed by aggregation of iso-tellurazole *N*-oxides in solution was assessed with dispersion- and gradient-corrected relativistic density-functional-theory (DFT) gas-phase calculations. For computational expediency, the models were based on the molecular building block with *R*=Me, **1c**. Calculations for the individual molecule were also used to build a map of electrostatic potential (Fig. 5a), which demonstrate the occurrence of two σ holes on the Te atom opposite to the C and N atoms, the latter hole being the most prominent. The LUMO of **1c** has a predominant contribution from the σ*_Te–N_ orbital (Fig. 5b). The plot of the electron localization function in the molecular plane (Fig. 5c) features two dips in the space surrounding the chalcogen. These data confirm that the molecules of **1** are predisposed to associate through contacts between the electrophilic (O) and nucleophilic (Te) regions and emphasize that the most favourable position for attachment of a Lewis base to is opposite to the nitrogen atom.

There is only one published^53^ crystallographic determination of an iso-selenazole *N*-oxide (3-methyl-phenyl-, **2b**), which features a centro-symmetric dimer formed by a pair of antiparallel Se^**…**^O interactions. The analogous structure for **1c**_2_, could only be optimized by imposing symmetry constraints. In their absence, the geometry converges to a dimer bridged by only one Te^**…**^O interaction (*d*=2.40 Å) in which the two heterocycles define an inter-planar angle of 87.6°, which is consistent with all the observed structures of the aggregates of **1a** and **1b**. The structures of the cyclic **1c**_4_ tetramers (chair and boat conformations) and the hexamer **1c**_6_ were also optimized, in each case vibrational calculations return all real frequencies confirming that all are minima in the potential-energy surface. Structures of hypothetical trimer and pentamer cyclic aggregates could not be satisfactorily optimized, the preferred nearly perpendicular orientation of the iso-tellurazole rings imposes a preference for an even number of molecules in a cyclic aggregate. Periodic calculations used to optimize the infinite chain **1c**_**∞**_, based on the structure observed in the crystal of 3(**1b**)·(C_6_H_6_). The results of these calculations are summarized in Table 1 as thermodynamic parameters for aggregation equilibria.

### Coordination of a transition-metal ion

**Figure 6:**
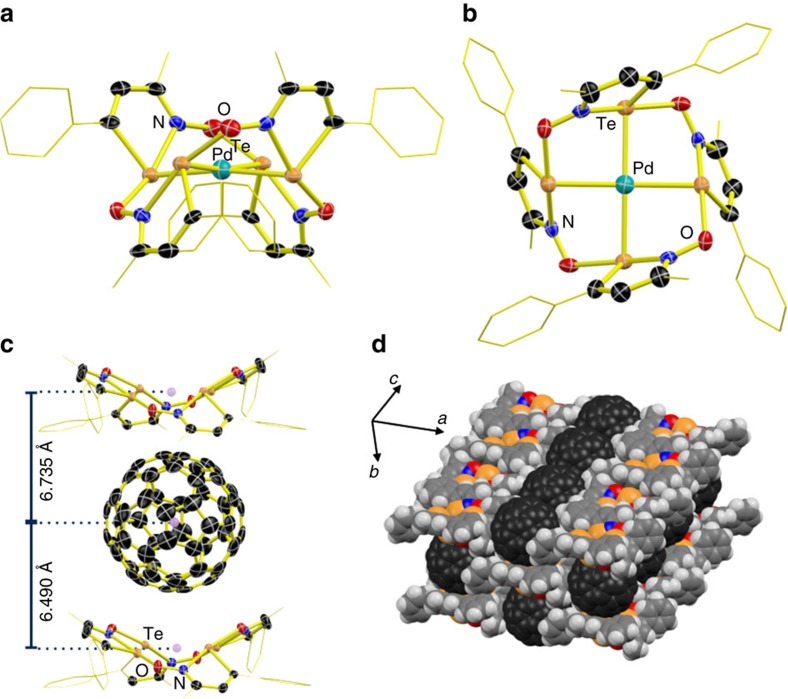
Detail of the crystal structures of the derivatives of the macrocyclic tetramer 1b_4_. (**a**,**b**) ORTEP perspectives of the [Pd(**1b**_4_)]^2^^+^ complex along (0,1,0) and along (2,1,0), respectively. (**c**) ORTEP of the crystal structure of **1b**_4_·C_60_. All displacement ellipsoids are shown at 75% probability. (**d**) Space-filling depiction of molecular packing the same crystal.

Mixing [Pd(NC-CH_3_)_4_](BF_4_)_2_ with **1b** dissolved in a CH_2_Cl_2_/acetonitrile mixture yields a dark brown mixture, its visible absorption spectrum features a well-defined shoulder at 500 nm. Job's continuous variations method showed that this spectrum is due to a complex of 1:4 stoichiometry (Fig. 1), the composition of which was confirmed by a structural determination from crystals grown by slow diffusion of an acetonitrile solution of the metal salt into a CH_2_Cl_2_ solution of **1b**. Figure 6a,b displays two views of the structure of the coordination complex in the crystal of [Pd(**1b**_4_)](BF_4_)_2_·2(CH_2_Cl_2_)_2_. The crystal structure features the tetrameric aggregate of **1b** in the boat conformation while the metal centre displays a square planar coordination geometry with Pd–Te distances (2.5804(4)Å) that are comparable to those measured in complexes of anionic tellurium ligands^55^; the Te–Pd–Te trans bond angles of 172.38(2)° denote a slight pseudo D_2d_ distortion. This crystal structure features metal depletion due to partial occupation of the coordination sites. CH_2_Cl_2_ molecules replaced the tetrafluoroborate anions in proportion to the missing metal ions. After refinement, the final ratios of occupancies from two crystals grown in separate batches, 0.863(7) and 0.797(5), are different as expected for independently prepared samples.

### C_60_ adduct

Mixing **1b** with C_60_ in chloroform immediately yields a solid that is not soluble enough for spectroscopic investigations. However, slow diffusion of C_60_ into a solution of **1b** produces crystals of composition 4(**1b**)·C_60_ (Fig. 1). Along the *b*-axis, the crystal structure features stacks of alternating fullerene molecules and distorted boat conformers of the **1b**_4_ aggregate (Fig. 6c). Compared with the boat **1a**_4_, in the adduct crystal the iso-tellurazole heterocycles are tilted towards the meridional plane of the macrocycle to maximize their contact with the fullerene. The stack is not symmetrical, there are two distinct distances between the centroids defined by the 4 tellurium atoms of each **1b**_4_ aggregate and the fullerene molecule, the closest macrocycle engaged in two short Te^**…**^C contacts (3.457(4) Å, *cf.*, the sum of van der Waals radii 3.76 Å) with the fullerene. The C_60_ molecule is slightly distorted, it features three crystallographically distinct diameter values (6.952(5), 6.9393(5) and 6.9223(5) Å), which evokes a Jahn–Teller distortion that would result from electron transfer into the t_1u_ LUMO^56^. However, there are no significant changes in the bond distances and angles of **1b**_4_ in this structure and the material is diamagnetic. As compared with the other crystalline phases in this report, the main differences are in the torsion angles only. Along *c*, the C_60_ molecules are organized in a columnar arrangement with even C^**…**^C spacing of 3.496 Å (Fig. 6d). The distance between C_60_ centroids is 10.533(2) Å, longer than the 10.008 Å observed in the crystal of pure C_60_ (ref. 57), and may be determined by the size of the macrocycle.

## Discussion

The variety of supramolecular structures obtained from **1b** and the ease with which the crystallization conditions select the aggregate and polymorph clearly indicate that these assemblies undergo reversible dissociation in solution. On the other hand, the short Te^**…**^O distances in the crystals structures and the results of mass spectrometry, NMR spectroscopy and DFT calculations show that the interaction between the tellurium and oxygen atoms is very strong.

The observation of aggregates in the electrospray mass spectrum **1b** is remarkable; supramolecular dimers assembled by Te^**…**^N interactions have been observed in mass spectra acquired from the laser-ablation plume of benzotelluradiazoles^58^ but the detection of oligomers with aggregation numbers 3–7 is unprecedented for organo-tellurium molecules.

Multinuclear NMR spectroscopic experiments demonstrate that the annular tetra- and hexamers are persistent and exist in equilibrium in solution. Direct observation of σ-hole interactions in solution is usually difficult^59^,^60^ but encapsulation of halogen-bonded adducts in cavitands^61^,^62^ has been helpful and in some cases can be monitored by spectroscopic methods^63^. Earlier observations of broadening in the ^1^H-NMR spectrum of **1a** at low temperature hinted at the existence of a dynamic process but in that instance the ^125^Te resonance could not be located and the nature of the process could not be conclusively established. The observation of NOE is one of the strongest evidences of the association of iso-tellurazole *N*-oxide molecules in solution at low temperature. Such spin cross-relaxation is only observable when the distance between the interacting nuclei is <5 Å, which is not possible within individual molecules of **1a** or **1b**. Moreover, that the NOE is negative indicates the zero quantum path is dominant in these systems, such situation is characteristic of restricted mobility due to large molecular weights, high viscosity and—arguably—cyclic structures. Provided there is no significant difference in the association energies of these molecules, the combination of **1a** and **1b** in solution would result in an even distribution of mixed structures that could be identified by their NMR spectra. For instance, an equimolar mixture that only forms centro-symmetric dimers would yield three different structures and display four lines from the methyl protons and two from the *t*-butyl groups; more complex patterns would arise from the dimers (Me: 8, *t*-Bu: 4), the tetramers (Me: 16, *t*-Bu: 8) and hexamers (Me: 41, *t*-Bu: 26). Of course, those are the maximum number of lines that would arise in each case; whether each of those lines could actually be observed would depend on the actual separation of their resonance frequencies, as well as on the dispersion and resolution provided by the instrument. The experimental result from the 1:1 mixture (Me: 14, *t*-Bu: 6; Fig. 4e) points to the tetramers, the size of these aggregates is confirmed by the observation of the mixed 1:3 and 2:2 macrocycles in the continuous variations experiments (Fig. 4f). Furthermore, the study of the concentration dependency of the ^1^H-NMR of **1a** at low temperature is consistent with the equilibrium between tetramers and hexamers in solution (Supplementary Fig. 3).

The thermodynamic parameters calculated with DFT-D3 for the model compound **1c** are indeed favourable for supramolecular association; however, their magnitudes are taken with caution as it has been argued that this method overestimates the binding energies of this type of interaction due to delocalization error. Also, solvation is likely to have an important role but these calculations do not account for it nor the effect of packing in a crystalline lattice. The calculations indicate that the binding energies of the Te^**…**^O interactions are nearly additive, there is little strain in the annular structures and only a small energetic difference between the two tetramer conformations. By enthalpy alone the hexamer would be the most stable cyclic structure, although entropy favours the smaller aggregates and individual molecules. Even more enthalpically favourable would be the infinite polymer chain but its formation naturally imposes the highest entropic cost.

The annular aggregates of iso-tellurazole *N*-oxides not only are persistent in solution but also display properties of actual macrocycles. The crystal structures of the hexamers already showcase their ability to host small molecules and suggest the construction of rotaxanes and inclusion compounds. Here we further demonstrate that they form coordination complexes and act as fullerene receptors.

The cyclic arrangement of chalcogen atoms and the trans-annular Te–Te distances of 5.0–5.6 Å suggest that the tetramers would be suitable to host transition-metal ions, this is indeed the case with Pd(II). The formation of the macrocyclic complex [Pd(**1b**_4_)]^2+^ is particularly significant; while the reversibility of the Te^**…**^O interactions favours the discreet oligomeric aggregates as the predominant species in solution, the lack of kinetic stabilization could compromise the structural integrity of the macrocycle. Iso-tellurazole oxides are potentially ambidentate ligands, coordination by oxygen would likely compete with the Te^**…**^O interactions. Such complication is possible even with soft metal ions; pyridine oxides, for example, easily coordinate palladium(II)^64^. As a macrocyclic ligand, the tetramer will enable the study of metal ions in a uniquely soft coordination sphere, which is difficult to achieve using more traditional approaches. Indeed, metal complexes of telluracrown ethers are difficult to obtain because their Te–C bonds are very reactive^65^.

Fullerenes form adducts with a variety of macrocyclic and polycyclic molecules in solution and are amenable to structural characterization by X-ray diffraction^66^. In the case of **1b**, poor solubility restricted the study of the product of reaction with C_60_ to the crystallographic determination but the ability of the tetramer **1b**_4_ to bind the fullerene receptor is well-demonstrated. The fullerene adduct is an intriguing material in its own right; its columnar arrangement of C_60_ molecules could facilitate charge transport, which calls for further investigations of applications in photovoltaics and molecular semiconductors.

As shown here, iso-tellurazole *N*-oxides have an unparalleled ability to spontaneously assemble functional macrocycles and thus hold great promise as supramolecular building blocks.

## Methods

### Experimental

The manipulation of air-sensitive materials was carried out in a glove box or using standards Schlenk techniques under an atmosphere of UHP argon (Praxair). Photosensitive materials were handled under a red LED illumination source. Elemental tellurium (CERAC), DMF (EMD), sodium hydroxide (EMD), acetic anhydride (Sigma-Aldrich), boron trifluoride diethyl etherate (Sigma-Aldrich), Boron trifluoride diethyl etherate (Sigma-Aldrich), hydroxylamine-*O*-sulfonic acid (Sigma-Aldrich), sodium borohydride (Sigma-Aldrich), tetrakis(acetonitrile)palladium(II) tetrafluoroborate (Sigma-Aldrich), Fullerene C_60_ (Sigma-Aldrich), dimethylcarbamoyl chloride (Alfa Aesar), phenylacetylene (Alfa Aesar), *t*-butylacetylene (Alfa Aesar), Chloroform (Caledon), dichloromethane (Caledon), diethyl ether (Caledon), ethyl acetate (Caledon), methanol (Caledon), Sodium sulphate (Caledon), toluene (Caledon), *n*-butyllithium (Acros Organics), Silica gel 60 (VWR) and sodium carbonate (VWR) were used as received from the commercial suppliers without further purification. Solvents for used synthesis were dehydrated within an Innovative Technologies solvent purification system (THF, acetonitrile) or by reflux with an appropriate dehydrating agent (Methanol over magnesium). 4-Phenylbut-3-yn-2-one and 5,5-dimethyl-hex-3-yn-2-one were prepared by literature methods. All NMR spectra were acquired in solution with a deuterated solvent. Spectra were obtained using Bruker AVANCE 500 MHz (Bruker 5-mm Broad Band Inverse probe) or Bruker AVANCE 600 MHz (Bruker 5-mm BROAD BAND OBSERVE probe) Spectrometers at 287.5 K unless otherwise indicated. Variable temperature spectra were acquired using either a cold or ambient temperature gas flow with a BV-T 2000 variable temperature controller. The sample temperature in the spectrometers was calibrated with a chemical-shift thermometer consisting of a 4% solution of methanol in methanol-d_4_. The ^1^H, ^13^C and ^125^Te spectra were processed using Bruker TopSpin 2.1 or 3.2 software packages. The ^1^H and ^13^C spectra were referenced to tetramethyl silane using the deuterated solvent signal as a secondary reference. The ^125^Te chemical shifts are reported respect to the room-temperature resonance of TeMe_2_ (*δ*=0.00 p.p.m.) but were measured using a secondary reference of diphenyl ditelluride in CD_2_Cl_2_ (*δ*=420.36 p.p.m.). Electrospray ionization mass spectra were acquired in positive ion mode on a Waters/Micromass Quattro Ultima Global ToF mass spectrometer operating in W Mode. Pure samples were dissolved in dichloromethane followed by dilution with methanol. High resolution Mass spectra were obtained in a Waters Global and Ultima (ES Q-TOF) Mass Spectrometer (capillary=3.20 V, cone=100 V, source temp=80 °C and resolving power=10,000). Infrared vibrational spectra were acquired in a Bio-Rad FTS-40 FT-IR spectrometer or a Thermo Scientific Nicolet 6700 FT-IR spectrometer. Melting points were determined with Uni-Melt Thomas Hoover capillary melting point apparatus and are reported uncorrected. Combustion elemental analyses were carried out by Guelph Analytical Laboratories (Guelph, Ontario, Canada).

### Synthesis overview

The iso-tellurazole *N*-oxides were prepared by a method (Supplementary Fig. 4) that includes the trans addition of an *in situ*-generated tellurocarbamic acid to an ynone. The resulting enone undergoes condensation with hydoxylamine-*O*-sulfonic acid to introduce the nitrogen atom and the heterocycle is closed by hydrolysis of the intermediate product. The process yields DMF and sulfuric acid as by-products that are separated in an aqueous workup.

### Bis-(*N*,*N*-dimethylcarbamoyl)-ditelluride

Sodium hydrogen telluride was prepared *in situ* from elemental tellurium (1.58 g), anhydrous sodium borohydride (2.33 g, 5 eq.) and anhydrous deoxygenated DMF (70 ml) at 95 °C under argon in a single-piece glass vessel. Shortly after heating started, the tellurium began to dissolve into a dark red–purple solution. After about 1 h, all the tellurium was consumed and the mixture became a light yellow suspension. The NaTeH dispersion was cooled to room temperature with a water bath and dimethylcarbamoyl chloride (3.97 g, 3 eq.) was added by cannula under argon; the reaction mixture was then stirred at 95 °C. The light yellow slurry was removed from the heat and cooled to room temperature in a water bath. Argon was removed with vacuum, and oxygen was introduced into the apparatus at 1 atm and the slurry became dark brown after 1 h. About 700 ml of distilled water was added into the mixture with stirring, the brown slurry turned black and was extracted repeatedly with 70 ml of diethyl ether until the aqueous solution was no longer yellow. The yellow organic solution was washed with aqueous sodium carbonate followed by distilled water, then dehydrated with Na_2_SO_4_.The organic fraction was concentrated under vacuum until a dark yellow solid began to precipitate at room temperature. The mixture was placed in a freezer (−20 °C) to promote crystallization of the pure ditelluride. Yield: 68%; mp: 105–110 °C (decomposed); ^1^H-NMR (500 MHz, CDCl_3_): *δ* 3.11 (s, 3H), 3.08 (s, 3H) (cf. ref. 66 3.11 (s, 3H), 3.08 (s, 3H)); ^13^C NMR (500 MHz, CDCl_3_): *δ* 145.4, 40.6, 36.1; IR (KBr): 1,660, 1,353, 1,249, 1,076, 872, 665 cm^−1^.

### (Z)-4-[(dimethylamino)carbonyltelluro]-4-phenyl-3-buten-2-one

Based on the procedure from the study by Shimada *et al*.^53^, bis(*N*,*N*-dimethylcarbamoyl)-ditelluride (1.54 g, 3.86 mmol) was dissolved in 35 ml of anhydrous DMF under argon; the solution was dark yellow. Anhydrous NaBH_4_ (0.321 g, 8.49 mmol) was dissolved in anhydrous methanol (18 ml) then added dropwise into the ditelluride solution while maintaining the temperature between −50 and −78 °C. The mixture was stirred at 0 °C for 30 min, the evolution of gas was observed and the colour of the mixture became dark red. The 4-phenylbut-3-yn-2-one (1.95 g, 13.51 mmol) was added to the reaction at 0 °C dropwise. The solution turned light yellow and stirring continued for 3 h. The reaction was quenched with 5 ml of distilled water followed by extraction with toluene in 50 ml portions from a 500 ml brine solution. The organic solution was washed with distilled water, dried with Na_2_SO_4_ and evaporated under high vacuum at 35 °C. The organic residue was purified by silica gel column chromatography with CH_2_Cl_2_:ethyl acetate (95:5% v/v). The solvent from eluate was evaporated and the product was a yellow solid. Yield: 72%; mp: 70–71 °C; ^1^H-NMR (500 MHz, CD_2_Cl_2_): *δ* 7.35–7.65 (m, 5H), 2.77 (s, 3H), 2.60 (s, 3H), 2.34 (s, 3H) (cf. ref. 53 7.33–7.41 (m, 5H), 2.81 (s, 3H), 2.60 (s, 3H), 2.56 (s, 3H)); ^13^C-DEPTq NMR (500 MHz, CD_2_Cl_2_): *δ* 197.2, 160.7, 156.6, 143.9, 131, 129.17, 128.9, 128.2, 41.7, 33.9, 30.1.

### (Z)-4-[(dimethylamino)carbonyltelluro]-4-*t*-butyl-3-buten-2-one

This derivative was synthesized in a similar way and obtained as a yellow solid. Yield: 37%; ^1^H-NMR (500 MHz, CDCl_3_): *δ* 6.81 (s, 1H), 3.00 (s, 6H), 2.28 (s, 3H), 1.25 (s, 9H); ^13^C-DEPTq NMR (500 MHz, CD_2_Cl_2_): *δ* 199.6, 156.9, 151.6, 133.4, 41.4, 35.9, 31.2, 30.7.

### 3-methyl-5-phenyl-1,2-tellurazole *N*-oxide, *R*=Ph (**1b**)

This compound was synthesized following the method described by Kübel^51^ with some modifications. The tellurocarbamate (1.42 g, 4.13 mmol) was refluxed with hydroxylamine-*O*-sulfonic acid (2.05 g, 18.16 mmol) for 1 h in anhydrous methanol (90 ml). The product was extracted with chloroform, washed with distilled water, dehydrated and dried under vacuum. The crude product was dissolved again in methylene chloride and was deposited on a layer of silica (2 cm). Impurities were eluted with methylene chloride through the silica. The pure product was then eluted with a CH_2_Cl_2_/methanol solution (50:50% v/v) and the solvent was removed under vacuum. The product was obtained as a pale yellow solid. Yield: >95%; mp: 207–211 °C (decomposed); ^1^H-NMR (500 MHz, CD_2_Cl_2_): *δ* 7.26–7.42 (m, 5H), 7.10 (s, 1H), 1.77 (s, 3H); ^13^C NMR (500 MHz, CD_2_Cl_2_): *δ* 157.9, 152.6, 140.8, 129.9, 128.2, 128.0, 127.7, 15.9; ^125^Te NMR (500 MHz, CD_2_Cl_2_): *δ* 1,595.2; IR (KBr): 3,050, 3,022, 2,918, 1,571, 1,493, 1,468, 1,443, 1,373, 1,343, 1,222, 1,109, 1,028, 927, 908, 869, 832, 759, 713, 696, 617, 584, 534 cm^−1^; HRMS (*m/z*): [M−H]^+^ calcd. for C_10_H_10_NOTe, 289.7961; found, 289.9831.

### 3-methyl-5-*t*-butyl-1,2-tellurazole *N*-oxide, *R*=*t*-Bu (1a)

This compound was synthesized in a similar way. Yield: 80%; mp: 180–185 °C (decomposed); ^1^H-NMR (500 MHz, CDCl_3_): *δ* 6.96 (s, 1H), 2.17 (s, 3H), 1.42 (s, 9H). ^13^C-DEPTq NMR (125.8 MHz, CD_2_Cl_2_): *δ* 168.9, 156.4, 122.7, 41.5, 32.1, 16.0; IR (KBr): 2,953, 2,912, 2,865, 1,565, 1,466, 1,424, 1,389, 1,370, 1,361, 1,337, 1,243, 1,231, 1,202, 1,125, 1,030, 1,001, 967, 896, 842, 828, 794, 760, 756, 697 cm^−1^; HRMS (*m/z*): [M-H]^+^ calcd. for C_8_H_14_ON^129^Te, 270.0138; found, 270.0122.

### [Pd(**1b**_**4**_)](BF_4_)_2_

A solution of isotellurazole-*N*-oxide in anhydrous dichloromethane (0.031 g, 0.108 mmol) was added dropwise to a solution of tetrakis(acetonitrile)palladium(II) tetrafluoroborate in anhydrous acetonitrile (0.012 g, 0.027 mmol). The solution turned from light yellow to deep red and a reddish–brown solid precipitated. The mixture was stirred under nitrogen for a day and the solid was filtered off, washed with dichloromethane and dried under vacuum. Yield: 98%; mp: 190–191 °C; ^1^H-NMR (500 MHz, CD_3_CN): *δ* 7.49-7.42 (m, 6H), 2.11 (s, 3H); ^13^C NMR (500 MHz, DMSO-d_6_): *δ* 138.4, 131.9, 129.2, 129.0, 128.7, 128.4, 128.1, 15.7; Not soluble enough for ^125^Te NMR; IR (KBr): 1,615, 1,575, 1,490, 1,442, 1,384, 1,219, 1,113, 1,084, 1,062, 928, 866, 760, 696, 614, 573, 533 cm^−1^; analysis (calcd., found for C_40_H_36_N_4_O_4_B_2_F_8_Te_4_Pd): C (33.66, 33.48), H (2.54, 2.28), N (3.93, 4.08). Slow diffusion in long tube yielded instead single crystals of idealized composition [Pd(**1b**_4_)](BF_4_)_2_.(CH_2_Cl_2_)_2_ in the mixing zone and crystals of pure **1b**_4_ at the bottom. The former loose the crystallization solvent under vacuum.

### [**1b**_**4**_]C_60_

A concentrated solution of **1b** (0.048 g, 0.167 mmol) was dissolved in chloroform. The layer of **1b** solution was allowed to diffuse with a layer concentrated solution of fullerene (0.030 g, 0.041 mmol) in tetrachloroethane that was filtered through an activated neutral alumina. Crystals suitable for X-ray diffraction were obtained by slow diffusion with the two layers of solution until the growth of crystals reached equilibrium. Yield: 76.9%; the material did not melt or appear to decompose up to 280 °C; [**1b**_**4**_]C_60_ is not soluble enough to acquire meaningful ^1^H, ^13^C and ^125^Te NMR spectra. IR (KBr): 1,572, 1,491, 1,468, 1,428, 1,372, 1,221, 1,182, 1,107, 1,028, 927, 908, 869, 833, 755, 715, 694, 616, 577, 527 cm^−1^; analysis (calcd., found for C_100_H_36_N_4_O_4_Te_4_): C (64.30, 64.16), H (1.94, 1.77 ), N (3.00, 2.75).

### Scrambling experiment

The NMR samples were prepared by mixing **1b** with **1a** in 10:0, 9:1, 8:2, 7:3, 6:4, 5:5, 4:6, 3:7, 2:8, 1:9, 0:10 molar ratios while maintaining a total amount of 8.717 × 10^−3^ mmol. Each sample was dissolved in 0.7 ml of deuterated methylene chloride, yielding a total concentration 12.6 mmol l^−1^. ^1^H-NMR spectra were acquired at both 179.9 and 287.5 K using a Bruker Avance 600 MHz spectrometer and are provided as Supplementary Fig. 5

### Single-crystal X-ray diffraction

Single crystals were grown under the following conditions: 3(**1b**)·(C_6_H_6_): slow evaporation from a benzene solution. **1b:** slow evaporation from a CH_2_Cl_2_ solution. 12(**1b**)·(CH_2_Cl_2_): slow evaporation from a concentrated solution in a mixture of or CH_2_CH_2_/pentane (90:10% v/v). 3(**1b**)·(C_4_H_8_O): slow evaporation from a THF solution. [Pd(**1b**_4_)](BF_4_)_2_(CH_2_Cl_2_)_2_: diffusion of a solution of **1b** in CH_2_Cl_2_ into [Pd(CH_3_CN)_4_])](BF_4_)_2_ in acetonitrile. (**1b**_**4**_)·**C**_**60**_: diffusion of a **1b** solution in CHCl_3_ into saturated C_60_ in tetrachloroethane. All crystals were mounted on a MiTeGen Micromounts with Paratone-n oil. Crystals were mounted on nylon loops (Hampton, CA) or MiTeGen Micromounts (Ithica, NY) with Paratone-n oil. A Bruker APEX2 diffractometer was used to collect data at 100 K with Mo-Kα radiation (*λ*=0.71073 Å). A CCD area detector was used and equipped with a low-temperature accessory Oxford cryostream. Solution and refinement procedures are presented in the Supplementary Methods and specific details are compiled in Supplementary Table 1. Selected distances and angles are provided in Supplementary Table 2.

### Computational

All DFT calculations were performed using the ADF/BAND DFT package (versions 2013 and 2014). Details of the method are provided in the Supplementary Methods. Coordinates of all optimized structures are provided in Supplementary Tables 3–8.

## Additional information

**Accession codes:** The X-ray crystallographic coordinates for all structures reported in this article have been deposited at the Cambridge Crystallographic Data Centre, under deposition numbers CCDC 1414076-1414081 and 1415229. These data can be obtained free of charge from The Cambridge Crystallographic Data Centre (www.ccdc.cam.ac.uk/data_request/cif).

**How to cite this article:** Ho, P. C. *et al*. Supramolecular macrocycles reversibly assembled by Te^…^O chalcogen bonding. *Nat. Commun.* 7:11299 doi: 10.1038/ncomms11299 (2016).

## Supplementary Material

Supplementary Information Supplementary Figures 1-5, Supplementary Tables 1-8, Supplementary Methods and Supplementary References

## Figures and Tables

**Table 1:** Calculated (PBE-D3) thermodynamic parameters of aggregation in gas phase.

**Equilibrium**	**Δ*****H*** **per Te**^**…**^**O interaction (kJ mol**^−**1**^**)**	**Δ*****S*** **per Te**^**…**^**O interaction (J mol**^−**1**^** K**^−**1**^**)**
2 **1c**⇌**1c**_2_	−68.7	−185
4 **1c**⇌**1c**_4_ (chair)	−68.6	−158
4 **1c**⇌**1c**_4_ (boat)	−69.3	−140
6 **1c**⇌**1c**_6_	−75.7	−171
∞ **1c**⇌**1c**_∞_	−82.1	−291
