# Supplementary material for: Supramolecular macrocycles reversibly assembled by Te…O chalcogen bonding
Source: Nat Commun. 2016 Apr 19;7:11299. doi: 10.1038/ncomms11299 (PMC4838886; doi:10.1038/ncomms11299)
Supplement: Supplementary Information — Supplementary Figures 1-5, Supplementary Tables 1-8, Supplementary Methods and Supplementary References [file ncomms11299-s1.pdf]

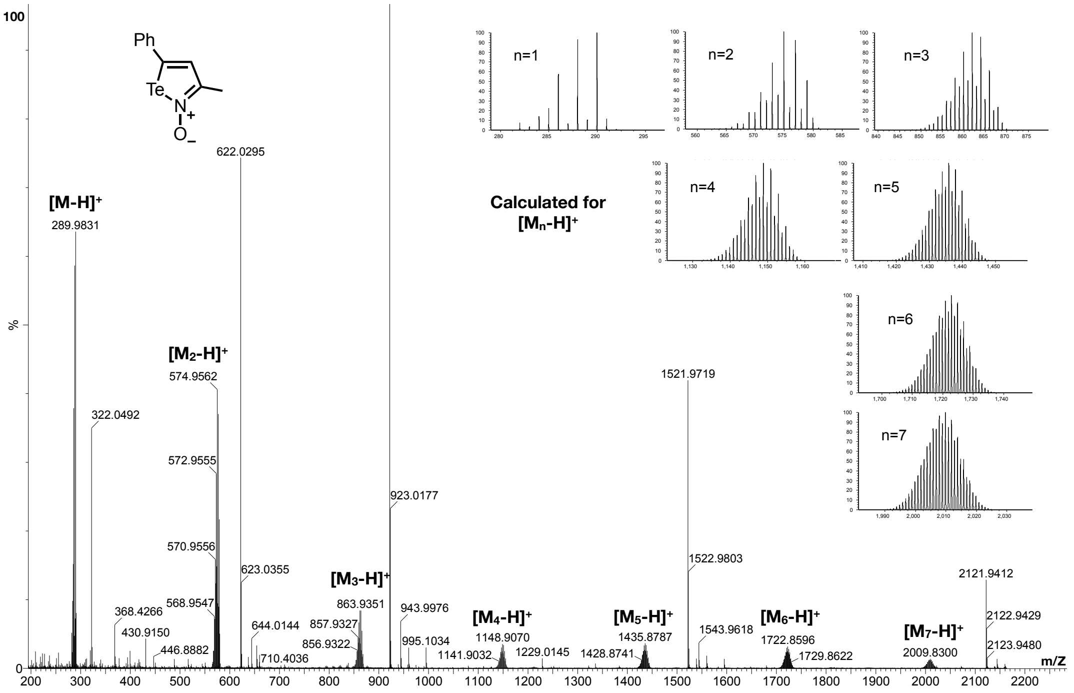

Supplementary Figure 1. Electrospray Mass Spectrum of **1b**. Calculated isotopic patterns for $[M^n\text{-}H]^+$ ions are displayed in the insets.

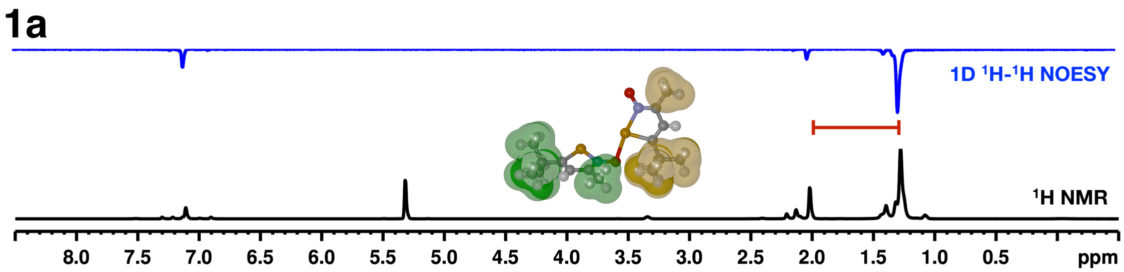

Supplementary Figure 2. 1D ^{1}H-NMR and 1D-NOESY spectra of **1a** in CD_2Cl_2 at 190K. The over-imposed structures portray the van-der-Waals surfaces of the methyl and *tert*-butyl groups of each molecule in a pair modeled with coordinates extracted from the crystal structures of $\mathbf{1a}_4$[1]. The resonance of residual $CHDCl_2$ is present at 5.32 ppm.

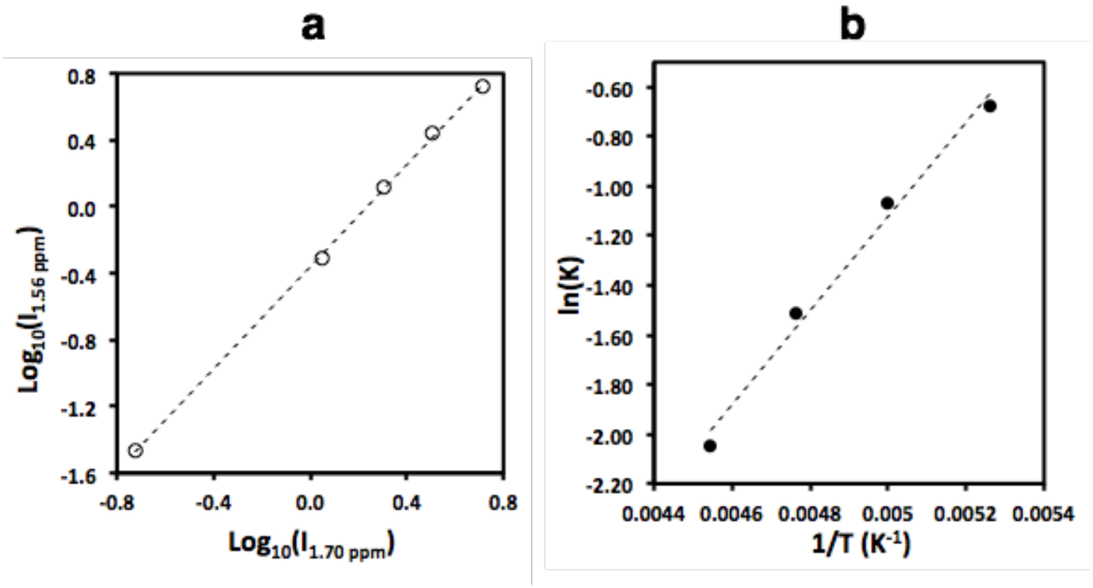

Supplementary Figure 3. Thermodynamics of aggregation of **1b**$_4$. a) Determination of stoichiometric coefficient (n) and equilibrium constant (K = |**1b**$_{4n}$ |/|**1b**$_4$|^n); b) van't Hoff plot.

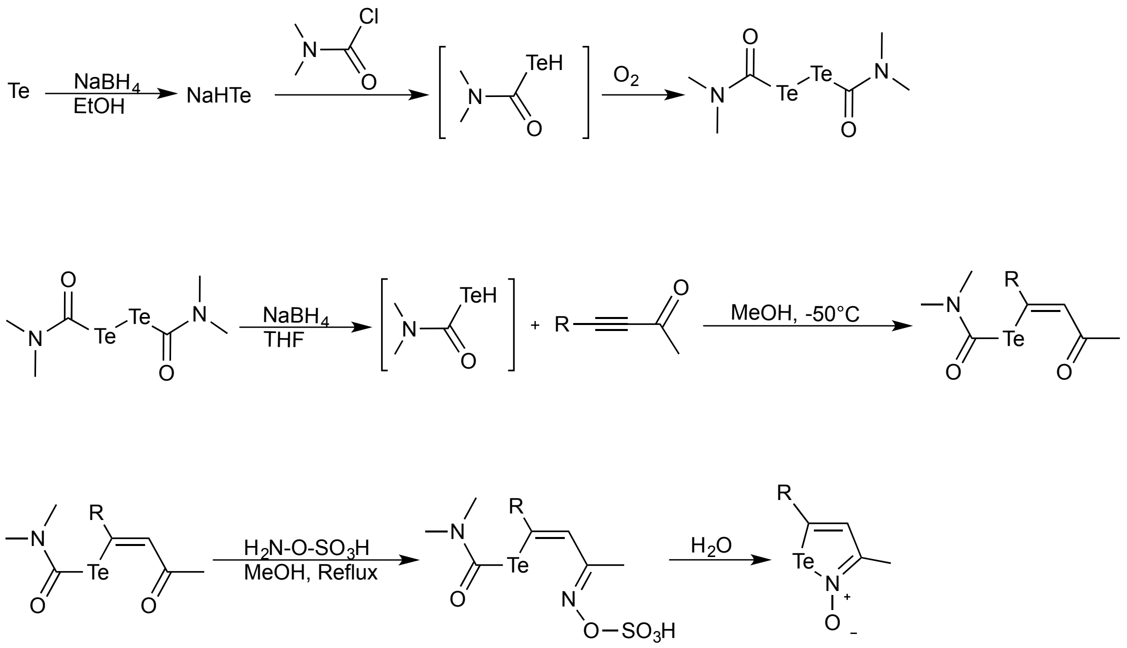

Supplementary Figure 4. Sequence of reactions used for the syntheses of **1a** (R=t-Bu) and **1b** (R=Ph).

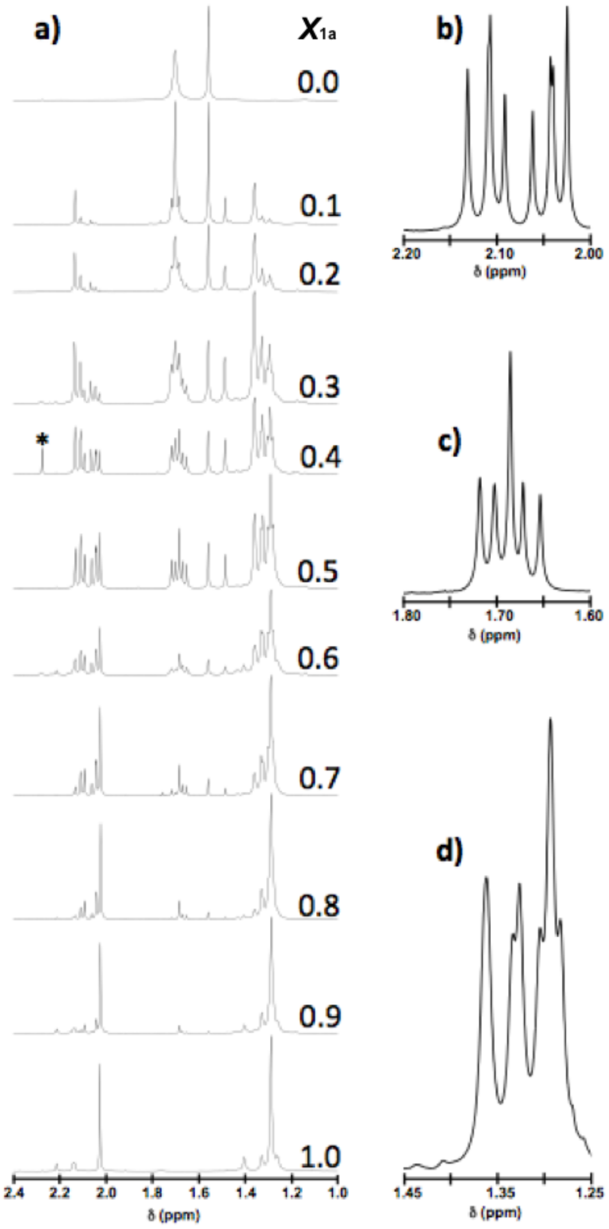

Supplementary Figure 5. a) ^{1}H NMR spectra of mixtures of **1a** and **1b** at 190 K, * denotes the resonance of a trace amount of THF. b), c), d) expanded sections of the spectrum of the equimolar mixture (i.e. $\boldsymbol{X}_{\mathbf{1a}}$ = 0.5).

Supplementary Table 1. Crystallographic and refinement parameters for all compounds.

**Crystal Composition**	3(**1b**)• (C_6H_6)	**1b**	12(**1b**)• 2(CH_2Cl_2)	3(**1b**)• (C_4H_8O)	0.86Pd•1.73BF_4)• 4(**1b**)• 1.72(CH_2Cl_2)	0.80Pd•1.59(BF_4)• 4(**1b**)• 1.41(CH_2Cl_2)	4(**1b**)•C_{60}
CCDC	1414076	1414079	1414078	1414081	1415229	1414080	1414077
Empirical formula	$C_{12}H_{11}NOTe$	$C_{10}H_9NOTe$	$C_{61}H_{55}N_6O_6ClTe$	$C_{68}H_{70}N_6O_7Te_6$	$C_{41.72}H_{39.44}B_{1.73}N_4O_4F_{6.92}Cl_{3.44}Pd_{0.86}Te_4$	$C_{41.41}H_{38.82}B_{1.59}N_4O_4F_{6.36}Cl_{2.82}Pd_{0.8}Te_4$	$C_{25}H_9NOTe$
Crystal system, Space group	Orthorhombic, P 2_1 2_1 2_1	Triclinic, P -1	Triclinic, P -1	Triclinic, R -3	Tetragonal, I4(1)/a	Tetragonal, I4(1)/a	monoclinic, C 2/c
a [Å]	8.6720(11)	8.3469(2)	14.457(3)	21.706(4)	11.2576(14)	11.2652(2)	23.9972(18)
b [Å]	12.6536(17)	9.7654(2)	15.311(3)	21.706(4)	11.2576(14)	11.2652(2)	13.2242(10)
c [Å]	31.538(4)	13.0143(3)	15.817(3)	12.127(2)	40.011(5)	40.0616(9)	21.0668(16)
α [°]	90	71.8880(8)	105.395(3)	90	90	90	90
β [°]	90	85.5200(8)	102.892(3)	90	90	90	97.8540(10)
γ [°]	90	82.0910(8)	103.246(3)	120	90	90	90
V [$Å^3$]	3460.7(8)	997.95(4)	3131.8(10)	4930.3(10)	5070.7(14)	5084.0(2)	6622.7(9)
Z, ρ(calc.) [g. cm^{-3}]	Z: 4	Z: 1, 1.915	Z: 2, 1.962	Z: 3, 1.8774	Z: 4, 1.966	Z: 4, 1.952	Z: 8, 1.934
T [K]	100.15	100.15	100.15	100(2)	100.15	100.15	296.15
μ[mm^{-1}]	2.551	2.939	3.188	2.677	2.777	2.760	1.815
θ range	2.436- 27.352	1.647- 26.371	2.357- 28.374	1.88- 27.46	1.879-25.346	1.878-27.499	4.781-34.974
Limiting indices	-11 <= h <= 9, -16 <= k <= 16, -37 <= l <= 40	-10 <= h <= 10, -12 <= k <= 12, -16 <= l <= 16	-18 <= h <= 18, -19 <= k <= 19, -19 <= l <= 19	-28 <= h <= 28, -28 <= k <= 23, -15 <= l <= 11	-13<= h <= 13, -7 <= k <= 13, -42 <= l <= 44	-14 <= h <= 14, -14 <= k <= 14, -52 <= l <= 52	-36 <= h <= 38, -21 <= k <= 18, -23 <= l <= 33
Refl. collec./ unique	38614/ 7931	27043/ 4082	53585/ 12799	9638/ 2522	9429/ 2277	32559/ 2929	31330/ 14335
R(int.)	0.0278	0.0158	0.0400	0.0264	0.0731	0.0261	0.0385
No. of parameters	409	237	736	93	189	189	507
R1* / wR2* (I>2σ(I))	0.0266/ 0.0554	0.0128/0.0315	0.0259/ 0.0516	0.0413/0.0942	0.0486/ 0.1306	0.0390/ 0.1288	0.0404/ 0.0793
R1* / wR2* for all data	0.0290/ 0.0575	0.0135/ 0.0318	0.0406/ 0.0553	0.0543 / 0.0998	0.0827/0.1428	0.0432/ 0.1309	0.0668/ 0.0862
Goodness-of-fit on F^2	1.159	1.080	0.995	0.9975	1.129	1.212	1.045
Larg. diff. peak/ hole [e.$Å^{-3}$]	1.206/ -0.702	0.957/ -0.303	0.970/ -1.074	2.623/ --2.091	1.465/ -0.832	2.730/ -0.884	1.363/ -0.745

- $R1 = \Sigma ||F_o|-|F_c|| / \Sigma|F_o|$, $wR2 = \{\Sigma[w(F_o^2-F_c^2)^2] / \Sigma w(F_o^2)^2\}^{½}$

Supplementary Table 2. Selected Structural Data

**Crystal Composition**	3(**1b**)•(C_6H_6)	**1b**	12(**1b**)•(CH_2Cl_2)	3(**1b**)•(C_4H_8O)	0.86Pd•1.73BF4)•<br>4(**1b**)•<br>1.72(CH2Cl2)	0.80Pd•1.59(BF4)•<br>4(**1b**)•<br>1.41(CH2Cl2)	4(**1b**)•C_{60}
**Aggregate**	**1b**$_\infty$	**1b**$_4$ (chair)	**1b**$_6$	**1b**$_6$	**1b**$_4$<br>(boat)	**1b**$_4$<br>(boat)	**1b**$_4$<br>(boat)
**Bond Distances (Å)**							
Te1-C1	2.100(5)<br>2.105(6)<br>2.106(6)	2.084(2)<br>2.080(2)	2.093(4)<br>2.099(3)<br>2.101(4)	2.096(5)<br>2.096(9)	2.12(1)	2.118(6)	2.088(2)<br>2.098(2)
Te1-N1	2.222(5)<br>2.241(5)	2.197(2)<br>2.198(1)	2.230(3)<br>2.257(3)<br>2.258(3)	2.246(4)<br>2.246(5)<br>2.246(6)	2.208(8)	2.207(5)	2.216(2)<br>2.225(2)
Te1···O1†	2.176(4)<br>2.207(4)<br>2.208(4)	2.203(1)<br>2.242(1)	2.171(3)<br>2.176(3)<br>2.178(2)	2.197(4)<br>2.197(6)	2.168(7)	2.174(5)	2.195(2)<br>2.221(2)
N1-O1	1.356(6)<br>1.357(6)<br>1.365(6)	1.360(2)<br>1.363(2)	1.359(4)<br>1.364(4)<br>1.366(3)	1.371(6)<br>1.371(7)<br>1.371(9)	1.37(1)	1.356(7)	1.356(3)<br>1.363(2)
N1-O1†	4.369(6)<br>4.399(6)<br>4.414(6)	4.355(2)<br>4.394(2)	4.370(4)<br>4.377(4)<br>4.407(4)	4.406(6)<br>4.406(7)<br>4.406(8)	4.33(1)	4.340(6)	4.372(3)<br>4.381(3)
C1-C2	1.345(8)<br>1.346(8)<br>1.352(9)	1.350(3)<br>1.357(2)	1.342(6)<br>1.343(6)<br>1.353(5)	1.34(1)<br>1.342(8)<br>1.342(9)	1.34(2)	1.343(9)	1.350(3)<br>1.351(4)
C2-C3	1.430(8)<br>1.434(8)<br>1.440(8)	1.433(3)<br>1.437(3)	1.435(5)<br>1.437(4)<br>1.445(6)	1.431(1)<br>1.431(8)	1.44(1)	1.45(1)	1.432(4)<br>1.434(4)
C3-N1	1.288(8)<br>1.298(7)<br>1.302(7)	1.303(2)	1.294(5)<br>1.298(4)<br>1.298(5)	1.312(7)	1.29(1)	1.295(8)	1.294(3)<br>1.308(3)
Te1···Te1†	3.7403(6)<br>3.7605(7)<br>3.7887(8)	3.8426(4)<br>3.8633(2)	3.6856(7)<br>3.7166(8)<br>3.7229(7)	3.8912(7)<br>3.8912(9)	3.6569(9)	3.6573(5)	3.6768(7)<br>3.7448(6)
**Bond Angles (°)**							
N1-Te1-O1†	165.9(2)<br>166.4(2)<br>166.6(2)	163.44(5)<br>163.52(5)	161.9(1)<br>166.2(1)<br>166.8(1)	165.2(2)	163.8(3)	163.8(3)	161.79(7)<br>163.22(7)
N1-Te1-C1	75.8(2)<br>76.0(2)<br>76.4(2)	76.36(6)<br>76.78(6)	75.2(1)<br>75.8(1)<br>75.9(1)	75.6(2)	75.6(3)	75.6(3)	76.40(8)<br>76.41(8)
O1-N1-Te1	125.8(3)<br>127.4(3)<br>127.8(3)	123.7(1)<br>126.2(1)	126.8(2)<br>127.6(2)<br>127.8(2)	126.5(3)<br>126.5(4)	123.6(6)	123.6(6)	127.2(1)<br>127.6(1)
**Torsion Angles (°)**							
Te1-N1-O1-Te1*	6.1(5)<br>14.5(5)<br>21.1(5)	63.1(1)<br>26.1(2)	4.9(3)<br>6.1(3)<br>20.7(3)	42.2(5)	41.5(7)	41.5(7)	10.2(2)<br>33.8(2)
N1-O1-Te1*-C1*	70.0(4)<br>86.4(4)<br>95.3(4)	101.5(1)<br>156.3(1)	64.7(2)<br>79.0(2)<br>95.4(2)	88.4(4)	107.8(6)	107.8(6)	108.4(2)<br>169.5(2)
C1-Te1-N1-O1	175.2(4)<br>177.6(5)<br>179.0(5)	174.0(1)<br>175.9(1)	175.7(3)<br>177.9(3)<br>178.3(3)	173.4(4)<br>173.4(5)	178.6(7)	178.6(7)	177.7(2)<br>178.9(2)

* and **†** denote chemically equivalent atoms in a neighboring **1b** molecule as defined in **Fig. 2**.

Supplementary Table 3. Optimized Cartesian coordinates of **1c.**

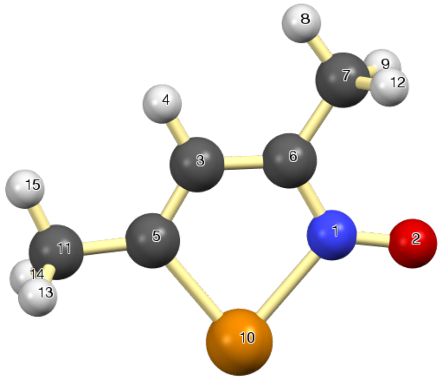

Atom	X	Y	Z
1.N	1.298052	0.016770	-0.747726
2.O	1.130421	-1.129482	-1.230082
3.C	2.155293	1.698897	0.681357
4.H	2.811007	1.961847	1.514882
5.C	1.418646	2.667344	0.064978
6.C	2.102797	0.333091	0.274293
7.C	2.876495	-0.784899	0.898356
8.H	3.504666	-0.408999	1.713509
9.H	3.510779	-1.280466	0.148673
10.Te	0.290296	1.813309	-1.466682
11.C	1.396598	4.123490	0.403455
12.H	2.195300	-1.554836	1.289918
13.H	0.391993	4.462391	0.697127
14.H	1.715624	4.747755	-0.444464
15.H	2.077789	4.322975	1.243116

Supplementary Table 4. Optimized Cartesian coordinates of **1c**$_2$.

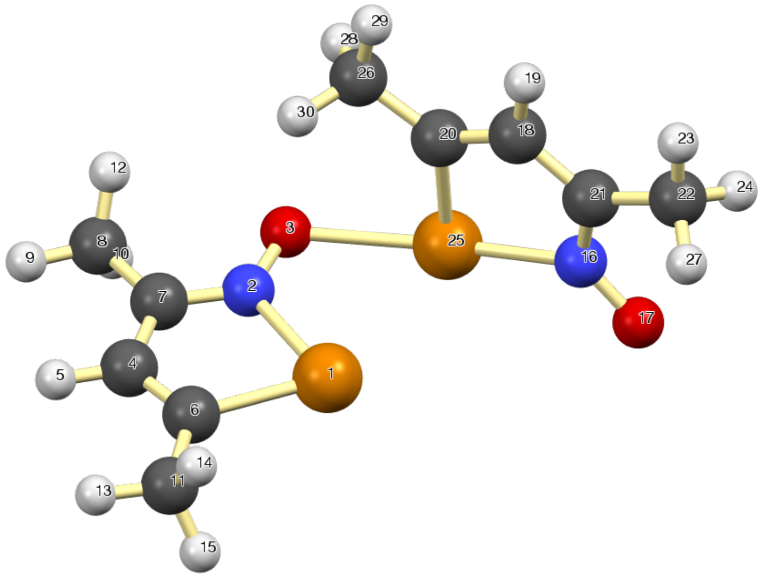

Atom	X	Y	Z
1.Te	1.388045	-0.908747	0.046879
2.N	1.527964	1.271069	-0.059200
3.O	0.518264	2.057759	0.206957
4.C	3.710639	0.722783	-0.674905
5.H	4.710455	1.049367	-0.971862
6.C	3.394953	-0.594037	-0.554500
7.C	2.717054	1.720912	-0.414174
8.C	2.936562	3.195878	-0.515233
9.H	3.962468	3.411895	-0.833396
10.H	2.747581	3.678749	0.454616
11.C	4.325533	-1.741025	-0.796374
12.H	2.230775	3.642992	-1.230138
13.H	5.316890	-1.376959	-1.101234
14.H	3.951810	-2.407328	-1.588009
15.H	4.454709	-2.356619	0.106128
16.N	-2.721950	-1.069376	0.751913
17.O	-2.972682	-1.800713	1.754372
18.C	-2.828179	-0.279893	-1.465233
19.H	-3.246384	-0.388174	-2.469673
20.C	-1.931303	0.712100	-1.218718
21.C	-3.250674	-1.214746	-0.461884
22.C	-4.225519	-2.327627	-0.679022
23.H	-4.590298	-2.331685	-1.712498
24.H	-5.079370	-2.230089	0.008154
25.Te	-1.329654	0.654500	0.799510
26.C	-1.398383	1.704097	-2.197741
27.H	-3.759250	-3.298690	-0.454107
28.H	-1.556216	2.739901	-1.865538
29.H	-1.897821	1.570364	-3.168295
30.H	-0.315442	1.581760	-2.347753

Supplementary Table 5. Optimized Cartesian coordinates of the chair conformer of **1c**$_4$.

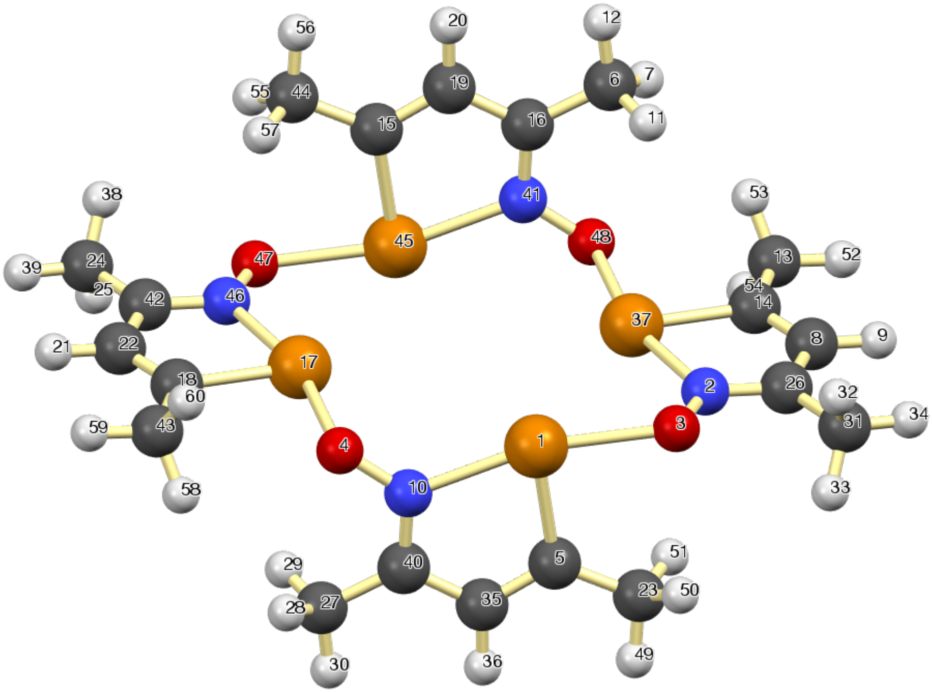

Atom	X	Y	Z
1.Te	1.110175	-2.622719	0.824099
2.N	2.197640	-1.038849	3.249799
3.O	2.059175	-2.319686	2.868617
4.O	-0.482847	-2.911626	-1.891843
5.C	3.045876	-2.597021	0.017269
6.C	-1.744977	2.964305	3.563379
7.H	-1.204316	3.880488	3.840639
8.C	2.928623	0.615493	4.708386
9.H	3.504731	0.905243	5.590270
10.N	0.733614	-2.838106	-1.337961
11.H	-1.178038	2.120305	3.984178
12.H	-2.744836	2.983654	4.010553
13.C	2.267256	3.022757	4.253081
14.C	2.270606	1.555420	3.969647
15.C	-3.045876	2.597021	-0.017269
16.C	-1.829826	2.846204	2.075382
17.Te	-1.269979	-0.746671	-2.313021
18.C	-2.270606	-1.555420	-3.969647
19.C	-3.053562	2.735279	1.340807
20.H	-3.997863	2.753747	1.889186
21.H	-3.504731	-0.905243	-5.590270
22.C	-2.928623	-0.615493	-4.708386
23.C	4.263504	-2.449510	0.868986
24.C	-3.521949	1.871761	-5.126283
25.H	-2.759242	2.576444	-5.489159

26.C	2.881668	-0.767945	4.345954
27.C	1.744977	-2.964305	-3.563379
28.H	1.204316	-3.880488	-3.840639
29.H	1.178038	-2.120305	-3.984178
30.H	2.744836	-2.983654	-4.010553
31.C	3.521949	-1.871761	5.126283
32.H	2.759242	-2.576444	5.489159
33.H	4.200831	-2.453718	4.486552
34.H	4.079502	-1.471626	5.980549
35.C	3.053562	-2.735279	-1.340807
36.H	3.997863	-2.753747	-1.889186
37.Te	1.269979	0.746671	2.313021
38.H	-4.200831	2.453718	-4.486552
39.H	-4.079502	1.471626	-5.980549
40.C	1.829826	-2.846204	-2.075382
41.N	-0.733614	2.838106	1.337961
42.C	-2.881668	0.767945	-4.345954
43.C	-2.267256	-3.022757	-4.253081
44.C	-4.263504	2.449510	-0.868986
45.Te	-1.110175	2.622719	-0.824099
46.N	-2.197640	1.038849	-3.249799
47.O	-2.059175	2.319686	-2.868617
48.O	0.482847	2.911626	1.891843
49.H	5.167714	-2.537982	0.250431
50.H	4.297191	-3.209262	1.660915
51.H	4.279712	-1.471757	1.372286
52.H	2.849845	3.232736	5.161362
53.H	1.245557	3.402359	4.388460
54.H	2.695013	3.597323	3.419824
55.H	-4.297191	3.209262	-1.660915
56.H	-5.167714	2.537982	-0.250431
57.H	-4.279712	1.471757	-1.372286
58.H	-1.245557	-3.402359	-4.388460
59.H	-2.849845	-3.232736	-5.161362
60.H	-2.695013	-3.597323	-3.419824

Supplementary Table 6. Optimized Cartesian coordinates of the boat conformer of **1c**$_4$.

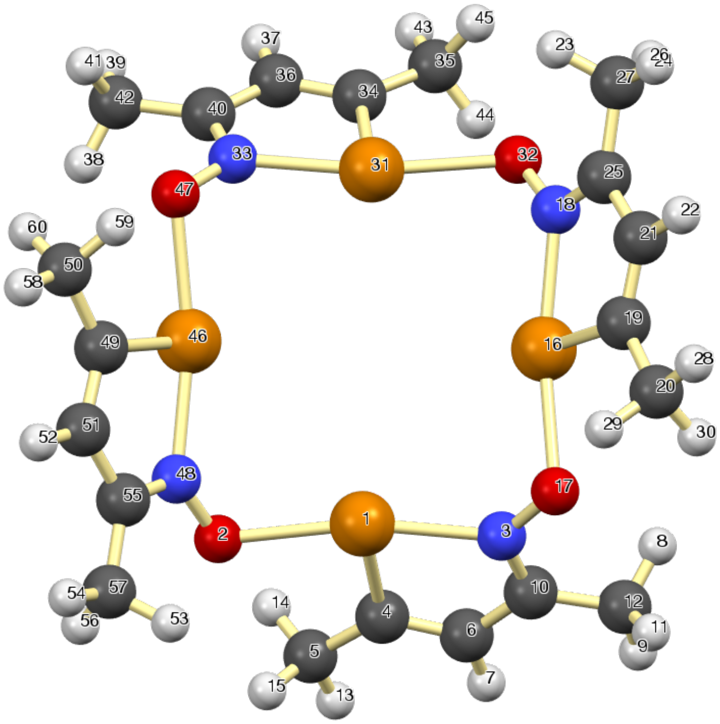

Atom	X	Y	Z
1.Te	1.420574	2.402288	0.139644
2.O	-0.410135	3.774302	0.546044
3.N	3.369054	1.403399	0.255944
4.C	2.330556	3.380150	1.744493
5.C	1.637628	4.455847	2.516269
6.C	3.591119	2.914259	2.001998
7.H	4.180673	3.334565	2.819420
8.H	5.426999	0.169038	1.550119
9.H	5.986083	1.689133	2.318897
10.C	4.150625	1.856301	1.222201
11.H	6.143550	1.433468	0.555011
12.C	5.505059	1.259331	1.433405
13.H	2.270873	4.791153	3.349823
14.H	0.677000	4.104250	2.916663
15.H	1.403531	5.320951	1.880036
16.Te	2.402288	-1.420574	-0.139644
17.O	3.774302	0.410135	-0.546044
18.N	1.403399	-3.369054	-0.255944
19.C	3.380150	-2.330556	-1.744493
20.C	4.455847	-1.637628	-2.516269
21.C	2.914259	-3.591119	-2.001998
22.H	3.334565	-4.180673	-2.819420
23.H	0.169038	-5.426999	-1.550119
24.H	1.689133	-5.986083	-2.318897
25.C	1.856301	-4.150625	-1.222201

26.H	1.433468	-6.143550	-0.555011
27.C	1.259331	-5.505059	-1.433405
28.H	4.791153	-2.270873	-3.349823
29.H	4.104250	-0.677000	-2.916663
30.H	5.320951	-1.403531	-1.880036
31.Te	-1.420574	-2.402288	0.139644
32.O	0.410135	-3.774302	0.546044
33.N	-3.369054	-1.403399	0.255944
34.C	-2.330556	-3.380150	1.744493
35.C	-1.637628	-4.455847	2.516269
36.C	-3.591119	-2.914259	2.001998
37.H	-4.180673	-3.334565	2.819420
38.H	-5.426999	-0.169038	1.550119
39.H	-5.986083	-1.689133	2.318897
40.C	-4.150625	-1.856301	1.222201
41.H	-6.143550	-1.433468	0.555011
42.C	-5.505059	-1.259331	1.433405
43.H	-2.270873	-4.791153	3.349823
44.H	-0.677000	-4.104250	2.916663
45.H	-1.403531	-5.320951	1.880036
46.Te	-2.402288	1.420574	-0.139644
47.O	-3.774302	-0.410135	-0.546044
48.N	-1.403399	3.369054	-0.255944
49.C	-3.380150	2.330556	-1.744493
50.C	-4.455847	1.637628	-2.516269
51.C	-2.914259	3.591119	-2.001998
52.H	-3.334565	4.180673	-2.819420
53.H	-0.169038	5.426999	-1.550119
54.H	-1.689133	5.986083	-2.318897
55.C	-1.856301	4.150625	-1.222201
56.H	-1.433468	6.143550	-0.555011
57.C	-1.259331	5.505059	-1.433405
58.H	-4.791153	2.270873	-3.349823
59.H	-4.104250	0.677000	-2.916663
60.H	-5.320951	1.403531	-1.880036

Supplementary Table 7. Optimized Cartesian coordinates of **1c**$_6$.

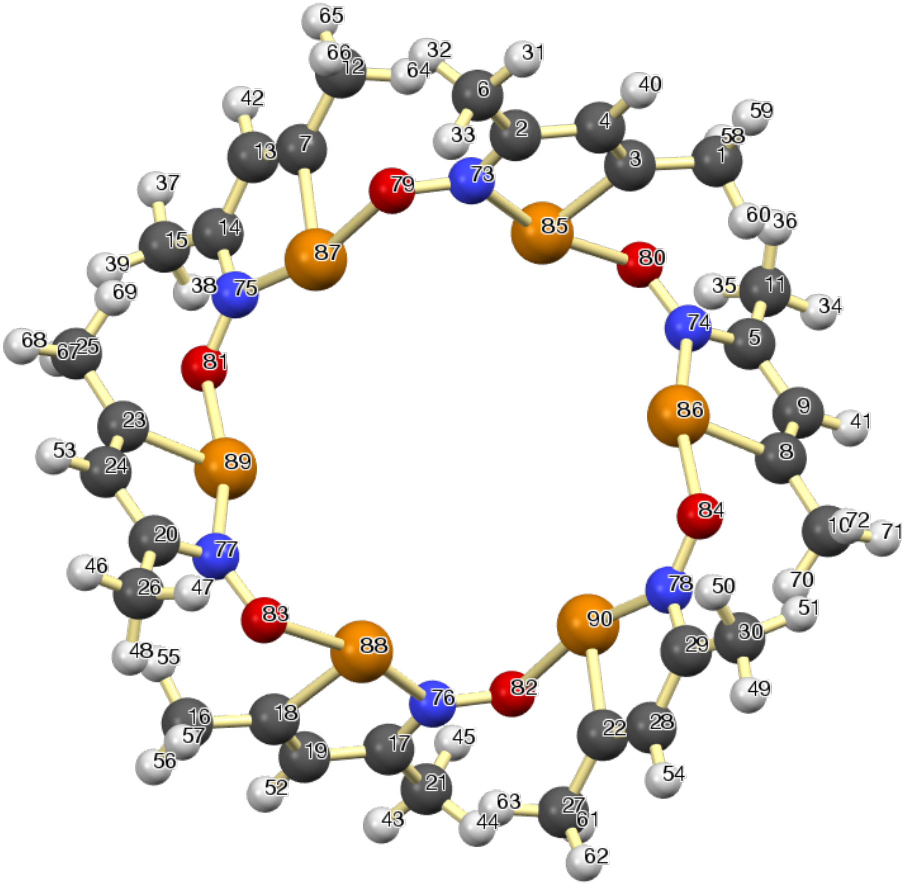

Atom	X	Y	Z
1.C	-4.620340	-4.351411	-0.373932
2.C	-5.046965	-1.043253	-2.278970
3.C	-4.551449	-2.926340	-0.816165
4.C	-5.172348	-2.425058	-1.919610
5.C	-1.619502	-4.892990	2.279167
6.C	-5.699896	-0.420626	-3.472040
7.C	-4.810546	2.479460	0.815986
8.C	0.258912	-5.405699	0.816284
9.C	-0.485567	-5.692562	1.919846
10.C	1.458661	-6.177904	0.374190
11.C	-2.484973	-5.146802	3.472459
12.C	-6.079018	1.826486	0.373538
13.C	-4.687171	3.267906	1.919403
14.C	-3.427905	3.850266	2.279055
15.C	-3.215507	4.727069	3.472166
16.C	4.620340	4.351411	0.373932
17.C	5.046965	1.043253	2.278970
18.C	4.551449	2.926340	0.816165
19.C	5.172348	2.425058	1.919610
20.C	1.619502	4.892990	-2.279167
21.C	5.699896	0.420626	3.472040

22.C	4.810546	−2.479460	−0.815986
23.C	−0.258912	5.405699	−0.816284
24.C	0.485567	5.692562	−1.919846
25.C	−1.458661	6.177904	−0.374190
26.C	2.484973	5.146802	−3.472459
27.C	6.079018	−1.826486	−0.373538
28.C	4.687171	−3.267906	−1.919403
29.C	3.427905	−3.850266	−2.279055
30.C	3.215507	−4.727069	−3.472166
31.H	−6.294042	−1.159325	−4.021524
32.H	−6.347859	0.412736	−3.163346
33.H	−4.942185	0.010151	−4.142466
34.H	−2.142234	−6.030565	4.022108
35.H	−2.479027	−4.275045	4.142660
36.H	−3.530726	−5.291323	3.163996
37.H	−4.152475	4.872291	4.021360
38.H	−2.463805	4.286274	4.142842
39.H	−2.817669	5.704890	3.163568
40.H	−5.783813	−3.072660	−2.552307
41.H	−0.230354	−6.545735	2.552735
42.H	−5.553900	3.473636	2.551890
43.H	6.294042	1.159325	4.021524
44.H	6.347859	−0.412736	3.163346
45.H	4.942185	−0.010151	4.142466
46.H	2.142234	6.030565	−4.022108
47.H	2.479027	4.275045	−4.142660
48.H	3.530726	5.291323	−3.163996
49.H	4.152475	−4.872291	−4.021360
50.H	2.463805	−4.286274	−4.142842
51.H	2.817669	−5.704890	−3.163568
52.H	5.783813	3.072660	2.552307
53.H	0.230354	6.545735	−2.552735
54.H	5.553900	−3.473636	−2.551890
55.H	3.639254	4.842849	0.457615
56.H	5.336114	4.902821	0.999778
57.H	4.925507	4.435497	−0.677377
58.H	−4.925507	−4.435497	0.677377
59.H	−5.336114	−4.902821	−0.999778
60.H	−3.639254	−4.842849	−0.457615
61.H	6.304209	−2.048625	0.677839
62.H	6.914588	−2.170678	−0.999179
63.H	6.014038	−0.731132	−0.457335
64.H	−6.014038	0.731132	0.457335
65.H	−6.914588	2.170678	0.999179
66.H	−6.304209	2.048625	−0.677839
67.H	−1.378876	6.484498	0.677040
68.H	−1.578406	7.073323	−1.000255
69.H	−2.374756	5.573884	−0.457687

70.H	2.374756	-5.573884	0.457687
71.H	1.578406	-7.073323	1.000255
72.H	1.378876	-6.484498	-0.677040
73.N	-4.299169	-0.317963	-1.473067
74.N	-1.873819	-3.882919	1.473066
75.N	-2.425706	3.565292	1.473377
76.N	4.299169	0.317963	1.473067
77.N	1.873819	3.882919	-1.473066
78.N	2.425706	-3.565292	-1.473377
79.O	-4.134826	0.986538	-1.754800
80.O	-2.921227	-3.088172	1.754834
81.O	-1.213866	4.075317	1.755148
82.O	4.134826	-0.986538	1.754800
83.O	2.921227	3.088172	-1.754834
84.O	1.213866	-4.075317	-1.755148
85.Te	-3.439865	-1.484229	0.245558
86.Te	-0.434374	-3.722263	-0.245776
87.Te	-3.005594	2.237859	-0.245308
88.Te	3.439865	1.484229	-0.245558
89.Te	0.434374	3.722263	0.245776
90.Te	3.005594	-2.237859	0.245308

Supplementary Table 8. Optimized Cartesian coordinates of **1c**$_\infty$ (1-D Lattice vector = 8.750435 Å).

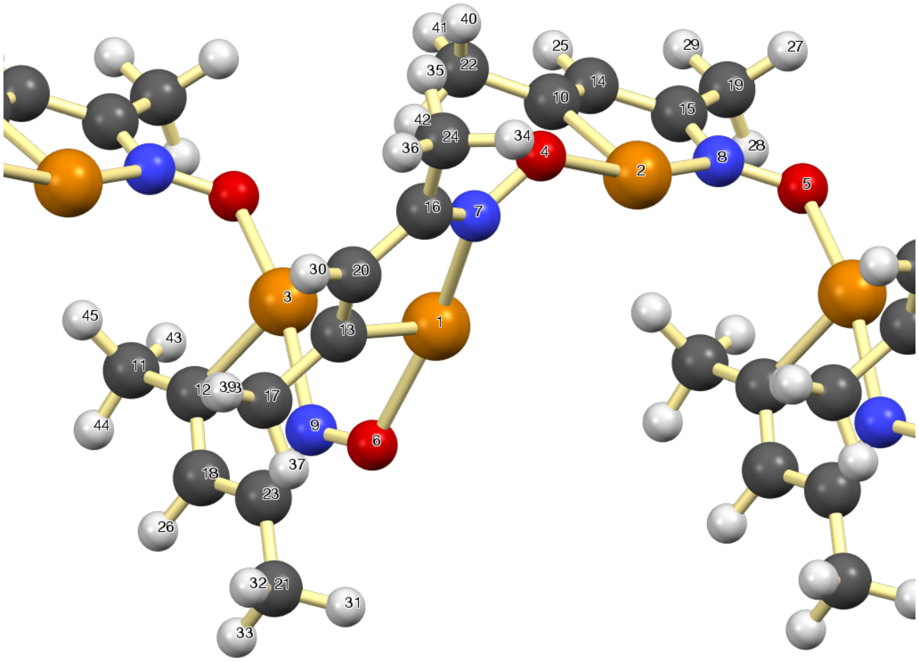

Atom	X	Y	Z
1.Te	3.821168	-0.752590	1.231537
2.Te	6.706353	1.347361	-0.032977
3.Te	0.913106	-0.609182	-1.210060
4.O	5.678606	1.853573	1.855642
5.O	8.638518	0.842652	-2.587565
6.O	2.647904	-2.583629	0.696520
7.N	4.730133	1.010169	2.293727
8.N	7.462529	1.316621	-2.147316
9.N	1.541631	-2.422246	-0.048867
10.C	5.184014	2.354405	-1.122515
11.C	-1.775475	-1.750563	-2.525897
12.C	-0.604357	-2.030701	-1.643058
13.C	2.757000	-0.655770	3.068294
14.C	5.465967	2.447345	-2.448872
15.C	6.687035	1.948565	-2.998245
16.C	4.247814	1.219399	3.498575
17.C	1.655419	-1.599963	3.428949
18.C	-0.405385	-3.222401	-1.020746
19.C	7.104057	2.153102	-4.413545
20.C	3.206987	0.322843	3.900749
21.C	1.014192	-4.742554	0.502951
22.C	3.930976	2.922246	-0.534000
23.C	0.735339	-3.451498	-0.189705
24.C	4.759714	2.359865	4.314073
25.H	4.751760	2.917008	-3.125489

26.H	-1.129942	-4.030020	-1.135341
27.H	8.049681	2.712963	-4.438935
28.H	7.284713	1.187637	-4.906431
29.H	6.326587	2.713908	-4.940118
30.H	2.776350	0.444653	4.897642
31.H	2.053705	-5.052097	0.332225
32.H	0.888443	-4.607660	1.587337
33.H	0.322642	-5.509657	0.141601
34.H	5.858265	2.360518	4.310888
35.H	4.447101	3.308116	3.850190
36.H	4.375049	2.300017	5.336682
37.H	1.995286	-2.643260	3.428296
38.H	1.272559	-1.363136	4.432887
39.H	0.814667	-1.537792	2.725007
40.H	4.143486	3.685734	0.225538
41.H	3.338042	3.395432	-1.331612
42.H	3.298569	2.159220	-0.059966
43.H	-1.457660	-1.444543	-3.531164
44.H	-2.406307	-2.646688	-2.615874
45.H	-2.401965	-0.942979	-2.125655

Supplementary Methods

DFT Calculations. All DFT calculations were performed using the ADF/BAND DFT package (version 2013).[2] Models for $\mathbf{1c}_n$ (n = 1, 2, 4, 6) were built from idealized internal coordinates and fully optimized using the exchange-correlation functional of Perdew, Burke, and Ernzerhof[3] and corrected for dispersion[4] with a triple-ζ all-electron basis set with two polarization functions each and applying the Zeroth Order Regular Approximation (ZORA)[5-8] formalism with specially adapted basis sets. Analytical vibrational frequencies [9-11] were calculated in order to demonstrate that each structure converged to an actual minimum in the potential energy surface and to derive the corresponding thermodynamic parameters. [12,13] Periodic calculations employed Becke's version of the fuzzy cells integration scheme. [14,15]

Crystallographic Solution and Refinement. Preliminary unit cell parameters were determined using a minimum of 50 frames from three different orientations, and final cell refinement after integration in SAINT.[16] Data were then corrected for absorption in SADABS.[17] The structures were solved by direct methods and refined by the full-matrix least-squares techniques on F^2 using SHELXL.[18] Anisotropic thermal parameters were assigned to all non-hydrogen atoms. Hydrogen atoms were placed in idealized positions using appropriate riding models. Specific details of each refinement are compiled in Supplementary Table 1.

Supplementary References

1. Kübel, J., Elder, P. J. W., Jenkins, H. A. & Vargas-Baca, I. Structure and formation of the first (–O–Te–N–)4 ring. *Dalton Trans* **39,** 11126–11128 (2010).
2. Guerra, C. F., Snijders, J. G., Velde, te, G. & Baerends, E. J. Towards an order-N DFT method. *Theor. Chem. Acc.* **99,** 391–403 (1998).
3. Perdew, J. P., Burke, K. & Ernzerhof, M. Generalized gradient approximation made simple. *Phys. Rev. Lett.* **77,** 3865–3868 (1996).
4. Grimme, S. Semiempirical GGA-type density functional constructed with a long-range dispersion correction. *J. Comp. Chem.* **27,** 1787–1799 (2006).
5. van Lenthe, E., Ehlers, A. & Baerends, E.-J. Geometry optimizations in the zero order regular approximation for relativistic effects. *J. Chem. Phys.* **110,** 8943–8953 (1999).
6. Van Lenthe, E., Baerends, E. J. & Snijders, J. G. Relativistic regular two-component Hamiltonians. *J. Chem. Phys.* **99,** 4597–4610 (1993).
7. Van Lenthe, E., Baerends, E. J. & Snijders, J. G. Relativistic total energy using regular approximations. *J. Chem. Phys.* **101,** 9783–9792 (1994).
8. Van Lenthe, E., Snijders, J. G. & Baerends, E. J. The zero-order regular approximation for relativistic effects: The effect of spin–orbit coupling in closed shell molecules. *J. Chem. Phys.* **105,** 6505–6516 (1996).
9. Bérces, A. *et al.* An implementation of the coupled perturbed Kohn-Sham equations: perturbation due to nuclear displacements. *Comp. Phys. Commun.* **100,** 247–262 (1997).
10. Jacobsen, H., Bérces, A., Swerhone, D. P. & Ziegler, T. Analytic second derivatives of molecular energies: a density functional implementation. *Comp. Phys. Commun.* **100,** 263–276 (1997).
11. Wolff, S. K. Analytical second derivatives in the Amsterdam density functional package. *Int. J. Quantum Chem.* **104,** 645–659 (2005).
12. and, M. S. & Bickelhaupt, F. M. Proton Affinities of Anionic Bases: Trends Across the Periodic Table, Structural Effects, and DFT Validation. *J. Chem. Theory Comput.* **2,** 281–287 (2005).
13. Swart, M., Rösler, E. & Bickelhaupt, F. M. Proton affinities of maingroup-element hydrides and noble gases: Trends across the periodic table, structural effects, and DFT validation. *J. Comp. Chem.* **27,** 1486–1493 (2006).
14. Becke, A. D. A multicenter numerical integration scheme for polyatomic molecules. *J. Chem. Phys.* **88,** 2547–2553 (1988).
15. Franchini, M., Philipsen, P. H. T. & Visscher, L. The Becke Fuzzy Cells Integration Scheme in the Amsterdam Density Functional Program Suite. *J. Comp. Chem.* **34,** 1819–1827 (2013).
16. Bruker (2012). *SAINT*. Bruker AXS Inc., Madison, Wisconsin, USA.
17. Bruker (2001). *SADABS*. Bruker AXS Inc., Madison, Wisconsin, USA.
18. Sheldrick, G. M. Crystal structure refinement with SHELXL. *Acta Cryst.* C**71**, 3-8 (2015).
